# COMMD10 inhibits tumor progression and induces apoptosis by blocking NF‐κB signal and values up BCLC staging in predicting overall survival in hepatocellular carcinoma

**DOI:** 10.1002/ctm2.403

**Published:** 2021-05-04

**Authors:** Mi Yang, Xixi Wu, Lu Li, Shaoqun Li, Nan Li, Mengyuan Mao, Suming Pan, Richang Du, Xiaoqing Wang, Min Chen, Nanjie Xiao, Xiaohui Zhu, Guoyang He, Longshan Zhang, Weiqiang Huang, Hua Pan, Lan Deng, Longhua Chen, Li Liang, Jian Guan

**Affiliations:** ^1^ Department of Radiation Oncology, Nanfang Hospital Southern Medical University Guangzhou Guangdong China; ^2^ Department of Radiotherapy Yue Bei People's Hospital of Guangdong province Shaoguan Guangdong China; ^3^ Department of Pathology Yue Bei People's Hospital of Guangdong province Shaoguan Guangdong China; ^4^ Department of Patholog, Nanfang Hospital Southern Medical University Guangzhou Guangdong China; ^5^ Department of Patholog, School of Basic Medical Sciences Southern Medical University Guangzhou Guangdong China; ^6^ Guangdong Province Key Laboratory of Molecular Tumor Pathology Guangzhou Guangdong China; ^7^ Department of Hematology, Zhujiang Hospital Southern Medical University Guangzhou Guangdong China

**Keywords:** Barcelona Clinic Liver Cancer (BCLC), copper metabolism MURR1 domain‐containing 10 (COMMD10), hepatocellular carcinoma, proliferation and apoptosis

## Abstract

**Background:**

Hepatocellular carcinoma (HCC) is the third leading cause of cancer mortality worldwide. Currently, there is limited knowledge of dysregulation of cellular proliferation and apoptosis that contribute to the malignant phenotype in HCC. Copper metabolism gene MURR1 domain 10 (COMMD10) is initially identified as a suppressor gene in the pathogenesis of HCC in our observations. Here we aimed to explore its function and prognostic value in the progression of HCC.

**Methods:**

Functional experiments were performed to explore the role of COMMD10 in HCC. The molecular mechanisms of COMMD10 were determined by luciferase assay, immunofluorescence, and immunoprecipitation. The nomogram was based on a retrospective and multicenter study of 516 patients who were pathologically diagnosed with HCC from three Chinese hospitals. The predictive accuracy and discriminative ability of the nomogram were determined by a C‐index and calibration curve and were compared with COMMD10 and the Barcelona Clinic Liver Cancer (BCLC) staging system. The primary endpoint was overall survival (OS).

**Results:**

COMMD10 expression was significantly lower in HCC than that in normal liver tissues. In vitro and in vivo experiments revealed that COMMD10 suppressed cell proliferation and induced apoptosis in HCC. Mechanistically, COMMD10 inhibits TNFα mediated ubiquitination of IκBα and p65 nuclear translocation through the combination of COMMD10‐N terminal to the Rel homology domain of p65, which inhibited NF‐κB activity and increased expression of cleaved caspase9/3 in HCC. Clinically, COMMD10 stratifies early‐stage HCC patients into two risk groups with significantly different OS. Additionally, the nomogram based on COMMD10 and BCLC stage yielded more accuracy than BCLC stage alone for predicting OS of HCC patients in three cohorts.

**Conclusions:**

COMMD10 suppresses proliferation and promotes apoptosis by inhibiting NF‐κB signaling and values up BCLC staging in predicting OS, which provides evidence for the identification of potential therapeutic targets and the accurate prediction of prognosis for patients with HCC.

## Graphical abstract

1. COMMD10 stabilizes IκBα protein by inhibiting ubiquitination and degradation of IκBα.

2. COMMD10 interacts with the Rel homology domain of p65 and suppresses the nuclear translocation of p65.

3. COMMD10/NF‐κB axis promotes apoptosis by modulating Bcl‐2/Bax/caspase‐9/3 pathway in hepatocellular carcinoma.

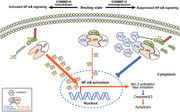

**ABBREVIATIONS**
ALB: albumin
BCLC: Barcelona Clinic Liver Cancer
CCK‐8: cell counting kit‐8
C‐index: concordance index
COMMD10: copper metabolism gene MURR1 domain 10
FFPE: formalin‐fixed paraffin‐embedded
FMNL2: formin‐like 2
HCC: hepatocellular carcinoma
IHC: immunohistochemical staining
IRS: immunoreactive score of Remmele and Stegner systems
OS: overall survival
PP: percentage of positive
ROC: receiver operating characteristic
SI: staining intensity

## 1 INTRODUCTION

Hepatocellular carcinoma (HCC) is the sixth most frequently diagnosed cancer and the third leading cause of cancer mortality worldwide, with an estimated 907,100 new cases and 821,700 deaths every year.^1^ Although various adjuvant therapeutic treatment of HCC is innovatory, the 5‐year survival rate of HCC is still less than 30%.^2^ Therefore, there is a pressing requirement to define the underlying molecular mechanisms during HCC tumor growth to develop novel therapeutic strategies. We previously demonstrated formin‐like 2 (FMNL2) as a tumor suppressor gene in HCC^3^ and further identified seven interacting proteins of FMNL2 by yeast two‐hybrid assay,^4^ such as COMMD10, DNAJA1, and SHANK2. COMMD10 is initially identified as a tumor regulator in the pathogenesis of multiple human tumors in our observations.^5^ Here we focus on COMMD10 to further refine the molecular mechanism of HCC development.

The transcription factor nuclear factor kappa B (NF‐κB), a central player critically involved in both inflammation and hepatocyte regeneration,^6^ is hyperactivated in HCC.^7^ HCC with high NF‐κB activity has uncontrolled inflammation accompanied by aggressive pathologic features and has poor treatment outcomes, whereas blockade of the NF‐κB pathway suppresses proliferation, demonstrating the essential role of NF‐κB activity inhibition in preventing HCC progression.^8^ COMMD (copper metabolism MURR1 domain‐containing) protein family members (COMMD1–COMMD10) have been shown to negatively regulate NF‐κB signaling in different ways,^9^ but the mechanism of NF‐κB in regulating HCC progression remains to be explored.

COMMD10, a member of the copper metabolism MURR1 domain‐containing protein family, has been found to participate in the control of several biological processes. COMMD10 was found to facilitate clearance of staphylococcus by promoting phagolysosomal maturation and acidification in infected liver kupffer cells.^10^ COMMD10 protects colon mucosa from systemic inflammation and inflammatory bowel disease by inhibiting the activation of Ly6C^hi^ monocyte of inflammasome.^11^ COMMD10 was also found to regulate the trafficking and ubiquitination of epithelial sodium channel by inhibition of Nedd4‐2, leading to Na^+^ transport alteration, which could be involved in the long‐term management of salt homeostasis and blood pressure.^12^ In addition, genome‐wide analysis showed that COMMD10 was a novel biologically relevant associated with systemic inflammation before fenofibrate treatment,^13^ and single nucleotide polymorphisms in the COMMD10 loci was associated with both asthma and chronic obstructive pulmonary disease.^14^ High‐throughput RNA sequencing identified COMMD10‐AP3S1 as a novel fusion transcript in colorectal cancer, which may aid the development of improved diagnostics and treatment.^15^ Moreover, our previous study elucidated the functions of COMMD10 in various human tumors based on expression profile and bioinformatics analysis, indicating its valuable role in the development and progression of tumor.^5^ Additionally, we found that COMMD10 inhibited the invasiveness and metastasis of colorectal cancer.^4^

To date, the role and mechanism of COMMD10 in HCC remain unknown. In this work, we investigated the role of COMMD10 in regulating cell proliferation and explored the underlying mechanisms and clinical values of COMMD10 in HCC.

## 2 MATERIALS AND METHODS

### 2.1 Cell lines and cell culture

HCC cell lines QGY‐7703, QGY‐7701, SMMC‐7721, and the normal hepatocyte cells HL‐7702 were purchased from Shanghai Institutes for Biological Sciences (China) and cultured in RPMI‐1640 medium (Biological Industries, USA) supplemented with 10% fetal bovine serum (Biological Industries, USA). HepG2 cells were maintained in high glucose DMEM (Biological Industries, USA). All the media were supplemented with 100μg/ml streptomycin and 100 U/ml penicillin (GibcoTM, Thermo Fisher Scientific, USA), and all cells were incubated at 37°C, 5% CO_2_.

### 2.2 Patients and tissue specimens

We used 516 formalin‐fixed paraffin‐embedded (FFPE) HCC specimens from three hospitals in China in this study. Three hundred ninety four samples pathologically diagnosed with HCC between January 2010 and December 2013 were collected from Nanfang Hospital of Southern Medical University, Guangzhou, China. Computer‐generated random numbers were used to assign these samples into a training cohort (NF training cohort) consisting of 260 samples and an internal validation cohort (NF internal validation cohort) consisting of 134 samples. Moreover, 61 samples collected from the Zhujiang Hospital between January 2010 and December 2013, and 61 samples collected from the Yuebei Hospital between August 2012 and December 2014 were used as external validation cohorts (ZY external validation cohort). The inclusion criteria were as follows: complete removal of macroscopic carcinomas; definitive histopathologic diagnosis of HCC in surgical samples; no preoperative anticancer treatments for HCC; and age ≥ 18 years. The exclusion criteria were as follows: recurrent HCC, tumors with mixed types on histopathologic analysis or of uncertain origin, perioperative mortality, and incomplete follow‐up data. In addition, we used GSE14520 dataset of GEO database as an external validation cohort.

### 2.3 Immunohistochemical staining and selection of cutoff point

Immunohistochemical staining (IHC) staining assay was performed on three‐micrometer‐thick FFPE sections. Briefly, sections were deparaffinized, hydrated, and boiled in sodium citrate buffer (0.01 M, pH 6.0) for 15 min and then incubated with COMMD10 antibody (Abcam, UK) at dilution of 1:200 (4°C, overnight), followed by incubation with the horseradish‐peroxidase‐conjugated anti‐goat secondary antibody (DakoCytomation, Glostrup, Denmark) for 1 h (37°C). The sections were stained with diaminobenzidine and counterstained in hematoxylin. The stained tissue sections were reviewed and scored by two pathologists blinded to the clinical parameters separately using immunoreactive score of Remmele and Stegner systems (IRS). COMMD10 staining was scored by multiplying the score of percentage of positive (PP) cells as 0 (no positive cells), 1 (<10% of percentage cells), 2 (11%–50%), 3 (51%–80%), 4 (> 80%), and the staining intensity (SI) as 0 (no color), 1 (mild reaction), 2 (moderate reaction), 3 (intense reaction). The products of SI and PP form an IRS ranging from 0 and 12 points. There are four grades of IRS: 0 = negative (0 or 1 point); 1 = positive, weak expression (2 or 3 points); 2 = positive, moderate expression (4–8 points) and 3 = positive strong expression (9–12 points).

The optimal cut‐off scores of COMMD10 were automatically selected based on Kaplan‐Meier analysis and log‐rank test by the X‐tile software.^16^ Firstly, we applied the X‐tile program software to generate an optimal value of COMMD10 staining cut‐off score to precisely classify patients according to clinical outcome in the training cohort. Then the value of COMMD10 staining cut‐off score was used to examine the correlation between COMMD10 expression and patients’ survival in validation cohorts.

### 2.4 Follow‐up and outcome

Postoperative follow‐up examinations were conducted at least every 3–6 months in the first 3 years and every 6–12 months thereafter. Follow‐up protocol was conducted on the recommendation of the European society of oncology.^17^ These include a detailed history, a complete physical examination, laboratory tests, and imaging examinations. The primary end point was overall survival (OS), which was calculated from the date of surgery to death. The data of patients without a documented OS event were censored at the last follow‐up.

### 2.5 Statistical analysis

The continuous variables were transformed into categoric variables according to the routine cut‐off points in clinical practice. Survival curves of patients were generated by the Kaplan–Meier method and the log‐rank test. When a variable reached a *p* value less than 0.05 in the univariate analysis, it was selected for multivariate Cox regression analysis.

The nomogram was established according to the results of multivariate Cox regression analysis in the NF training cohort. The final predictive model was selected via a backward step‐down selection process using the Akaike information criterion.^18^ The discriminatory ability of the nomogram was quantified by the C‐index and evaluated by comparing survival probability of nomogram‐predicted and observed Kaplan–Meier estimates, for which bootstrapping with 1000 resamples was applied. Comparison between the nomogram and Barcelona Clinic Liver Cancer (BCLC) stage was performed using the rcorrp.cens function of the Hmisc package in R version (http://www.r‐project.org/) and tested using the C‐index. The nomogram was also assessed in terms of area under the receiver operating characteristic (ROC) curve. The total score of each patient in the validation cohort was calculated in the light of the established nomogram, and then the total score was used as a factor for Cox regression analysis. Ultimately, the C‐index and calibration curve were obtained on the basis of regression analysis. The identification of risk factors was performed with SPSS software (IBM Corp., Armonk, USA). The rms package in R was used to establish the nomogram.

Data analysis was performed using the method of log‐rank test, Student's 2‐tailed *t*‐test, Fisher's exact test, and χ2 test. Data are reported as the mean ± SD, two‐sided, and *p* values of less than 0.05 were considered statistically significant.

## 3 RESULTS

### 3.1 COMMD10 disturbed the growth of HCC in vitro and in vivo by regulating the NF‐κB signaling pathway

**FIGURE 1:**
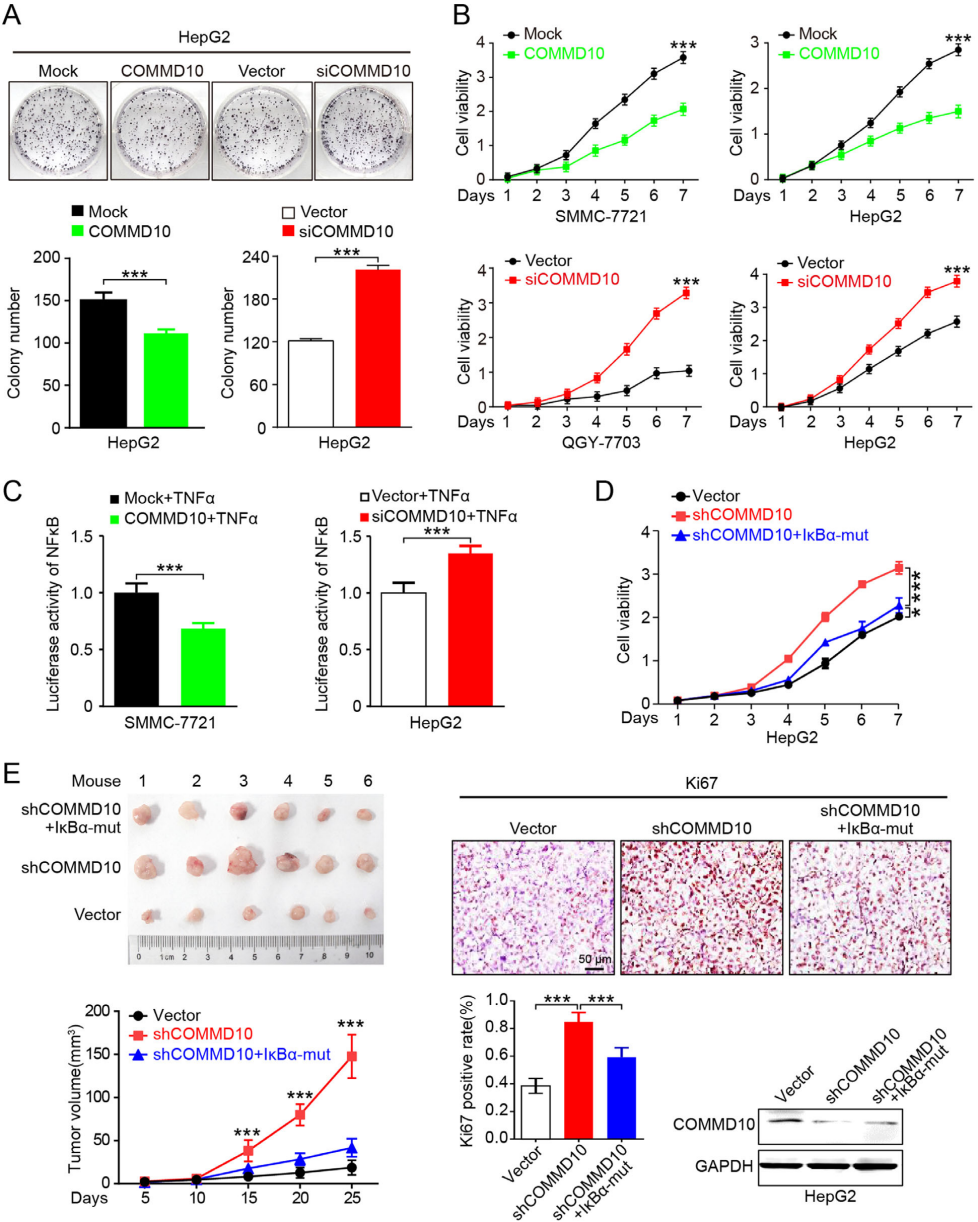
COMMD10 suppresses proliferation of HCC in vitro and in vivo. (A) Representative images of the clone formation assay (upper); the percentage of colonies was counted in graphs (bottom). (B) Growth curves for the indicated HCC cells (SMMC‐7721, HepG2, QGY‐7703) were evaluated by CCK‐8 assay. (C) NF‐κB luciferase‐reporter activity in COMMD10 overexpressing SMMC7721 and COMMD10‐deficient HepG2 cells under TNFα (10 ng/ml) treatment. (D) Overexpressing IκBα‐mut inhibited COMMD10‐deficiency induced proliferation as evaluated by CCK‐8 assay in HepG2 cells. (E) Tumor growth of HepG2 cells expressing vector or shCOMMD10 or shCOMMD10+IκBα‐mut in nude mice (*n* = 6, left). Representative images of IHC staining of the proliferation marker Ki67 in the indicated group, and quantification of the positive nuclei for this marker (*n* = 6, right). COMMD10 content of tumor xenografts in each group was detected by Western blot (right). GAPDH was used as loading control. Each bar represents the mean ± SD; *p < 0.05; **p < 0.01; ***p < 0.001

To investigate the potential role of COMMD10 in the development of HCC, we firstly examined COMMD10 expression in HL‐7702 normal hepatocytes and HCC cell lines (HepG2, Huh7, QGY‐7701, QGY‐7703, SMMC‐7721) (Figure S1A). The level of COMMD10 expression was significantly reduced in HCC cell lines compared to HL‐7702 cells. Then COMMD10 was overexpressed or depleted in the following function experiments according to the endogenous expression level (Figure S1B and S1C). Colony formation assay showed that overexpression of COMMD10 significantly inhibited while depletion of COMMD10 obviously increased the proliferation ability of HepG2 cells (both *p* < 0.001, Figure 1A). Consistently, cell counting kit‐8 (CCK‐8) assays of COMMD10‐overexpressing SMMC‐7721 and HepG2 cells revealed significantly decreased cell viability. Conversely, QGY‐7703 and HepG2 cells with COMMD10 knockdown had strongly elevated cell viability compared to that in control cells (all *p* < 0.001, Figure 1B).

We previously have predicted the potential regulation of NF‐κB by COMMD10 using bioinformatics analysis,^5^ thus we tested whether COMMD10 expression would be involved in cell proliferation of HCC via NF‐κB regulation. Luciferase activity assay showed that COMMD10 overexpression strongly inhibited tumor necrosis factor alpha (TNFα)‐mediated NF‐κB activation (*p* < 0.001, Figure 1C left). Conversely, NF‐κB was activated in COMMD10‐depleted cells (*p* < 0.001, Figure 1C right). These data suggest that COMMD10 can impair NF‐κB signaling, while silencing COMMD10 enhances NF‐κB activity in HCC. We further tested the effect of IκBα‐mut, a NF‐κB pathway inhibitor, on COMMD10 depletion‐induced HCC proliferation. As expected, ectopic expression of IκBα‐mut significantly decreased COMMD10 depletion‐induced cell viability (*p* < 0.001, Figure 1D). The capability of COMMD10 to suppress HCC progression was further examined using a xenograft tumor model. Tumors derived from HepG2/shCOMMD10 cells were larger and had higher Ki67 proliferation index compared to those in vector group (*p* < 0.001, Figure 1E). However, overexpression of IκBα‐mut could significantly decreased size and Ki67 proliferation index of tumors derived from HepG2/shCOMMD10 cells (*p *< 0.001, Figure 1E). In addition, COMMD10 was significantly decreased in tumors derived from both HepG2/shCOMMD10 and HepG2/shCOMMD10/IκBα‐mut cells compared to tumors derived from HepG2/vector cells (*p* < 0.001, Figure 1E). Collectively, the above findings verified that COMMD10 functions as a HCC suppressor through inhibiting NF‐κB signaling pathway.

### 3.2 COMMD10 inhibits TNFα‐mediated ubiquitination and degradation of IκBα and suppresses the NF‐κB signaling pathway

**FIGURE 2:**
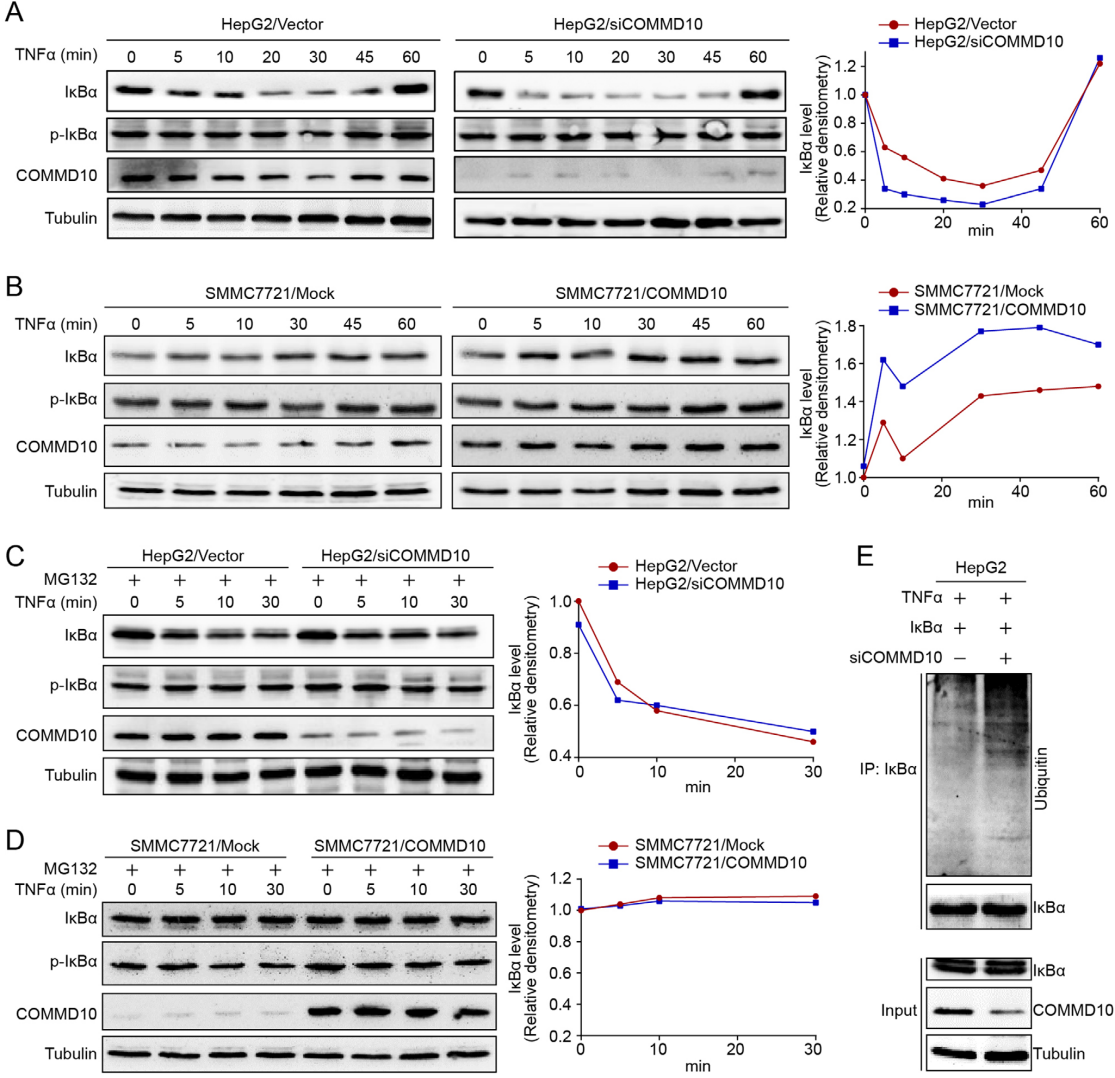
COMMD10 inhibits TNFα mediated ubiquitination and degradation of IκBα and suppresses the NF‐κB signaling pathway. (A) Effect of COMMD10 deficiency on the level of IκBα, p‐IκBα in HepG2/Vector and HepG2/siCOMMD10 cells exposed to TNFα (10 ng/ml) as illustrated. Line graph showed the gray scale value of IκBα at indicated time in HepG2/Vector and HepG2/siCOMMD10 cells. (B) Effect of COMMD10 overexpression on the level of IκBα, p‐IκBα in SMMC7721/Mock and SMMC7721/COMMD10 cells exposed to TNFα (10 ng/ml) as illustrated. Line graph showed the gray scale value of IκBα at indicated time in SMMC7721/Mock and SMMC7721/COMMD10 cells. (C) Effect of COMMD10 deficiency on the level of IκBα, p‐IκBα in HepG2/Vector and HepG2/siCOMMD10 cells exposed to MG132 (40μM) and TNFα (10 ng/ml) as illustrated. Line graph showed the gray scale value of IκBα at indicated time in HepG2/Vector and HepG2/siCOMMD10 cells. (D) Effect of COMMD10 overexpression on the level of IκBα, p‐IκBα in SMMC7721/Mock and SMMC7721/COMMD10 cells exposed to MG132 (40μM) and TNFα (10 ng/ml) as illustrated. Line graph showed the gray scale value of IκBα at indicated time in SMMC7721/Mock and SMMC7721/COMMD10 cells. (E) Effect of COMMD10 deficiency on the level of IκBα ubiquitination in HepG2/Vector and HepG2/siCOMMD10 cells exposed to TNFα (10 ng/ml)

To investigate the potential molecular mechanisms by which COMMD10 suppresses HCC proliferation through regulating NF‐κB activity, we examined the effect of COMMD10 overexpression or depletion on the expression of IκBα subunit. We found that COMMD10 deficiency affected neither the IκBα mRNA, the IκBα protein nor the phosphorylated IκBα (p‐IκBα) protein in HepG2 cells without TNFα stimulation (Figures S2A, S2B, and 2A). Similarly, we found that COMMD10 overexpression did not affect the expression level of IκBα and p‐IκBα protein in SMMC7721 cells without TNFα stimulation (Figure 2B). However, COMMD10 deficiency caused a faster decrease and lower expression of IκBα, and COMMD10 overexpression induced a higher expression of IκBα, which is independent on the IκBα phosphorylation (Figures 2A and 2B).

Considering that the degradation of IκBα is mediated by phosphorylation and subsequent ubiquitination‐proteasome degradation, we speculated that COMMD10 may regulate the ubiquitination of IκBα protein. To verify this, we detected the expression level of IκBα in HCC cells treated with proteasome inhibitor MG132 and found that IκBα protein was not affected by COMMD10 deficiency or overexpression compared to corresponding control cells (Figures 2C and 2D). Indeed, we found that COMMD10 deficiency significantly increased the level of ubiquitinated IκBα protein (Figure 2E). The above results indicate that COMMD10 inhibits TNFα‐mediated ubiquitination and degradation of IκBα and suppresses the NF‐κB signaling pathway.

### 3.3 COMMD10 interacts with RHD of p65 and suppresses the nuclear translocation of p65

**FIGURE 3:**
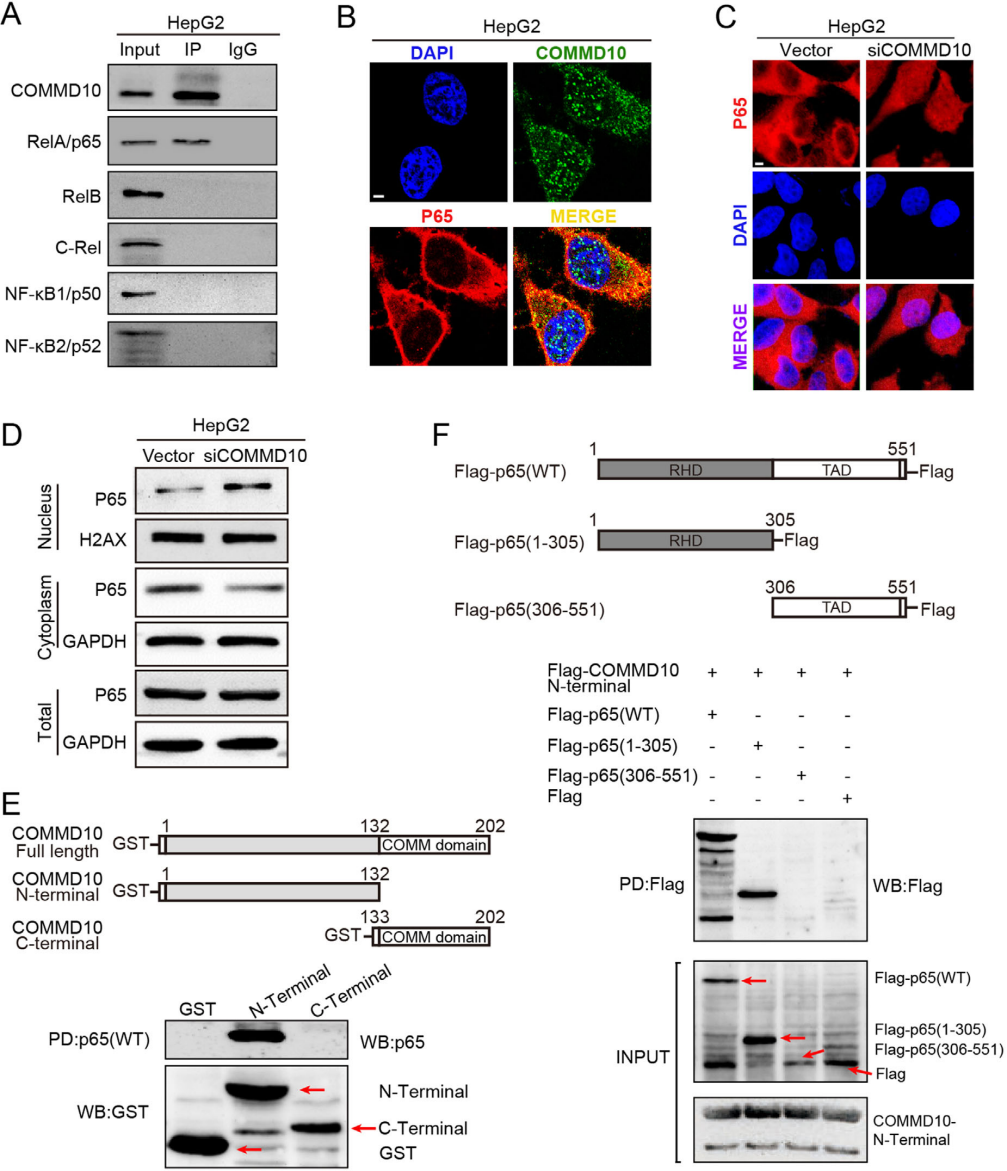
COMMD10 interacts with RHD of p65 and suppresses the nuclear translocation of p65. (A) Endogenous COMMD10 was immunoprecipitated (IP) from HepG2 cell lysates. (B) Immunofluorescence staining of COMMD10 and NF‐κB p65 colocalization in the cytoplasm. (C) Translocation of NF‐κB p65 in HepG2 cells by Immunofluorescence staining. (D) Western blot of nuclear, cytoplasmic, and total NF‐κB p65 expression in HCC cells. H2AX (H2AX histone family member) was used as the nuclear loading control; GAPDH was used as the cytoplasmic loading control. (E) Schematic structure of COMMD10 and its truncations (top) and GST‐Pull down assay showed domains of COMMD10 involved in the interaction with p65 (bottom). (F) Schematic structure of p65 and its truncations (top) and GST‐pull down assay showed domains of p65 involved in the interaction with COMMD10 (bottom). Abbreviation: PD, pull down.

The activation of the NF‐κB pathway depends on the nuclear translocation of homodimers or heterodimers, the most abundant form of which is the p65:p50 heterodimer via the canonical pathway.^19^ We further examined the effect of COMMD10 overexpression or depletion on expression levels of NF‐κB subunits. Unexpectedly, none NF‐κB subunit (i.e., p65, RelB, cRel, NF‐κB1/p50, NF‐κB2/p52) was changed (Figure S3A), indicating that COMMD10 inhibits NF‐κB signaling without altering the levels of subunits. The possibility that COMMD10 interacts with the NF‐κB complex was evaluated in HepG2 cells using co‐immunoprecipitation assays. COMMD10 interacted with p65, but not with other of NF‐κB subunits (Figure 3A). Immunofluorescence co‐localization assay showed that COMMD10 and p65 mainly co‐localized in the cytoplasm, and rarely in the nucleus (Figure 3B). Interestingly, p65 was significantly reduced in the cytoplasm and highly increased in the nucleus after COMMD10 expression was silenced, whereas total p65 was unchanged (Figures 3C and 3D). These data indicate that p65 accumulation in the nucleolus is a consequence of deficiency of endogenous COMMD10. To further identify the potential combination sites of p65 in COMMD10, C‐terminal (amino acids 1–132) and N‐terminal (amino acids 132–202) truncation mutants of COMMD10 were constructed (Figure 3E top). GST‐pulldown assay showed that N‐terminal of COMMD10 specifically combined with the wild type p65 (Figure 3E bottom). Moreover, the binding domain of p65 with the N‐terminal of COMMD10 was further investigated by constructing two truncation mutants of p65 (Figure 3F top): one composed of the Rel homology domain (RHD, amino acids 1–305) that includes the function of DNA binding, dimerization, and nuclear localization, and the other is a fusion of a C‐terminal region of p65 (amino acids 306–551) that includes the transcription activation domain. As expected, COMMD10 specifically bound to the wild type p65 and truncated mutant p65 (amino acids 1–305) (Figure 3F bottom). These data suggested that N‐terminal of COMMD10 combines with RHD of p65 and suppresses NF‐κB signaling pathway.

### 3.4 COMMD10/NF‐κB axis promoted intrinsic apoptosis by modulating Bcl‐2/Bax/caspase‐9/3 pathway

Previous studies have reported the regulation of apoptosis by NF‐κB signaling.^20^ Based on the inhibiting effect of COMMD10 on p65, we explored whether COMMD10 is involved in regulating apoptosis in HepG2 cells. Real‐time PCR revealed that the mRNA levels of known apoptosis‐related genes, including caspase‐3 (CASP3), CASP8, CASP9, and PARP1 (poly[ADP‐ribose] polymerase 1), were significantly decreased, while the anti‐apoptotic gene BCL2 was increased in HepG2/siCOMMD10 cells compared to HepG2/vector cells (*p* < 0.05, Figure S3B).

**FIGURE 4:**
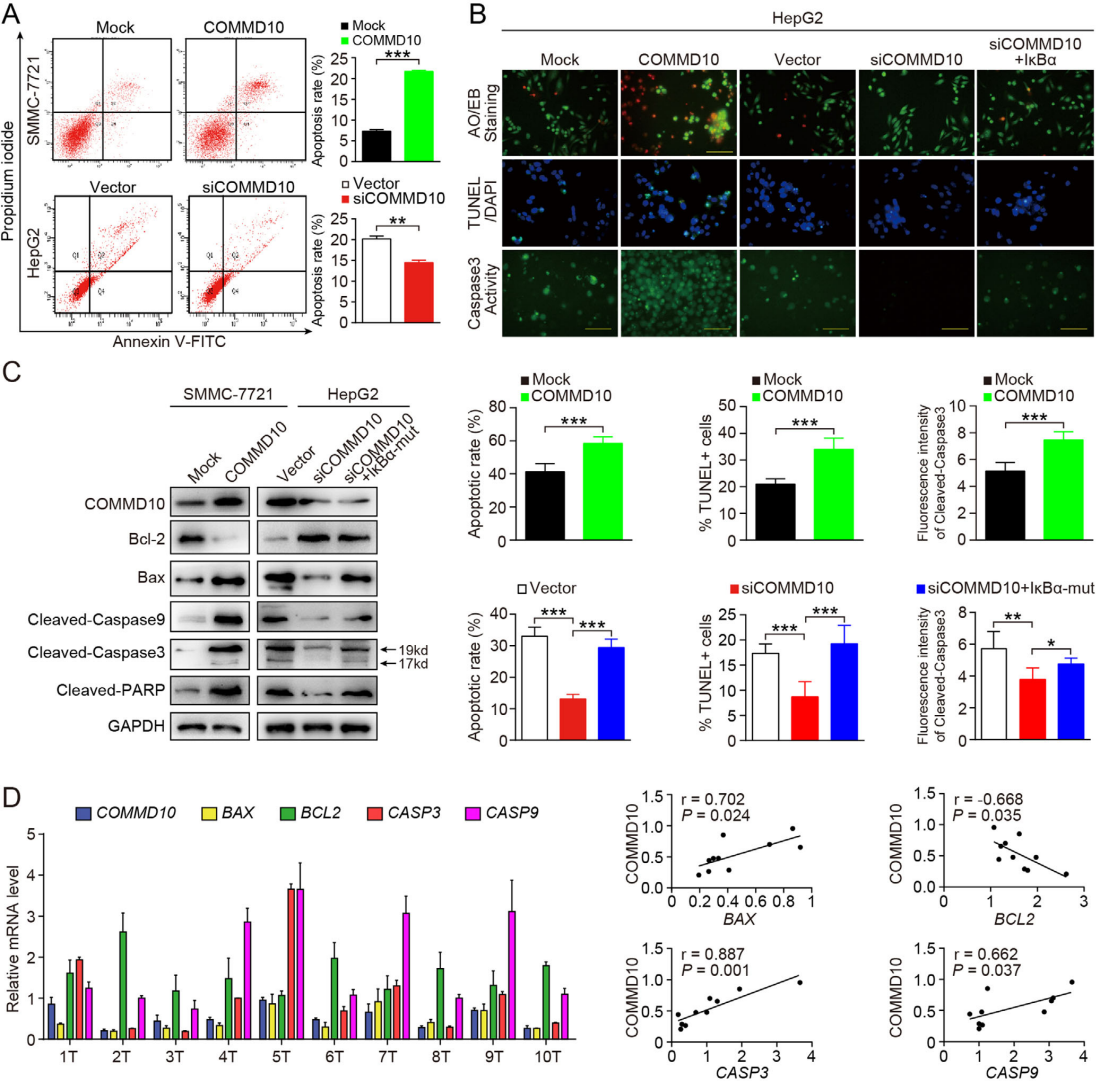
COMMD10/NF‐κB/Bcl‐2/Bax/Caspase9/3 axis associates with apoptosis. (A) Annexin V‐fluorescein isothiocyanate (FITC)/propidium iodide (PI) staining of indicated cells treated with irradiation (6 Gy). (B) A series of apoptosis assay induced by cisplatin (10ug/ml). Representative images of AOEB staining (upper), TUNEL assay (middle) and caspase3/7 activation assay (bottom) were showed from top to bottom, and the corresponding statistical analysis chart arranged from left to right. (C) Western blot of Bcl2/, Bax, cleaved‐caspase3, cleaved‐caspase9, and cleaved‐PARP expression in the indicated cells exposed to cisplatin (100μg/ml). GAPDH was used as a loading control. (D) Analysis of mRNA level (left) and correlation (right) of COMMD10 with BAX, BCL2, CASP3, and CASP9 in 10 freshly collected human HCC samples. The data are shown from a single representative experiment of three repeats. Each bar represents the mean ± SD; *p < 0.05; **p < 0.01; ***p < 0.001

The role of COMMD10 in apoptosis was further explored in vitro. Flow cytometry showed that overexpression of COMMD10 significantly increased while depletion of COMMD10 significantly decreased apoptotic rates compared to control cells in HCC (*p* < 0.001, Figure 4A). Consistently, AO/EB double staining indicated that the amount of apoptotic cells in COMMD10 expressing group was obviously more than that in mock group. On the contrary, depletion of COMMD10 significantly decreased the number of apoptotic cells compared to vector group. However, overexpression of IκBα‐mut could attenuate the inhibition effect of COMMD10 depletion on cell apoptosis in HepG2 cells (*p* < 0.001, Figure 4B, top). Table S1 summarizes the apoptosis rates in each group. Moreover, both TUNEL assay (*p* < 0.001, Figure 4B, middle) and the caspase‐3/caspase‐7 activation assay (*p* < 0.01, Figure 4B, bottom) yielded consistent results with the AO/EB double staining.

To investigate the underlying mechanism involved in the regulation of apoptosis, we investigated intrinsic apoptosis‐related proteins in SMMC‐7721/COMMD10 and HepG2/siCOMMD10 cells treated with cisplatin, radiation or TNFα. The result of western blot revealed that COMMD10 overexpression significantly reduced Bcl‐2 while enhanced Bax levels in HCC cells exposed to cisplatin. Some apoptosis proteins regulated by Bcl‐2/Bax were also correspondingly changed, such as cleaved CASP9, cleaved CASP3, and cleaved PARP (Figure 4C). Similarly, COMMD10 also increases the expression level of pro‐apoptotic proteins while inhibits the expression of BCL‐2 expression in irradiated or TNFα treated HCC cells (Figures S4A and S4B). Consistently, COMMD10 levels in freshly collected clinical HCC samples correlated positively with the mRNA levels of BAX (*r* = 0.702, *p* = 0.024), CASP3 (*r* = 0.887, *p* = 0.001), CASP9 (*r* = 0.662, *p* = 0.037), and negatively with BCL‐2 (*r* = ‐0.668, *p* = 0.035) (Figure 4D). We also found that extrinsic apoptosis pathway inducer cleaved CASP8 was elevated in COMMD10‐overexpressing HCC cells, but was downregulated in COMMD10‐silenced HCC cells. However, cleaved CASP8 expression was unchanged when NF‐κB activity was inhibited by IκBα‐mut, indicating that the apoptosis pathway involving CASP8 might not be a downstream target of the COMMD10/NF‐κB pathway (Figure S5A). The significant positive correlation between COMMD10 and CASP8 mRNA levels in the clinical samples provides further evidence that COMMD10 is functionally and clinically relevant to HCC cell apoptosis (Figure S5B). Accumulating evidence showed that IAP protein family members (XIAP, cIAP1 [baculoviral IAP repeat‐containing 2], cIAP2) downstream of NF‐κB can suppress different apoptotic pathways by inhibiting distinct caspases. However, XIAP and cIAP1 expression was unchanged in the COMMD10‐overexpressing and COMMD10‐silenced HCC cells (Figure S5A). Therefore, our findings validate that the COMMD10/NF‐κB axis promotes intrinsic apoptosis by modulating Bcl‐2/Bax/caspase‐9/3 pathway in HCC.

### 3.5 The relationship between COMMD10 expression and patient clinic‐pathologic features

**FIGURE 5:**
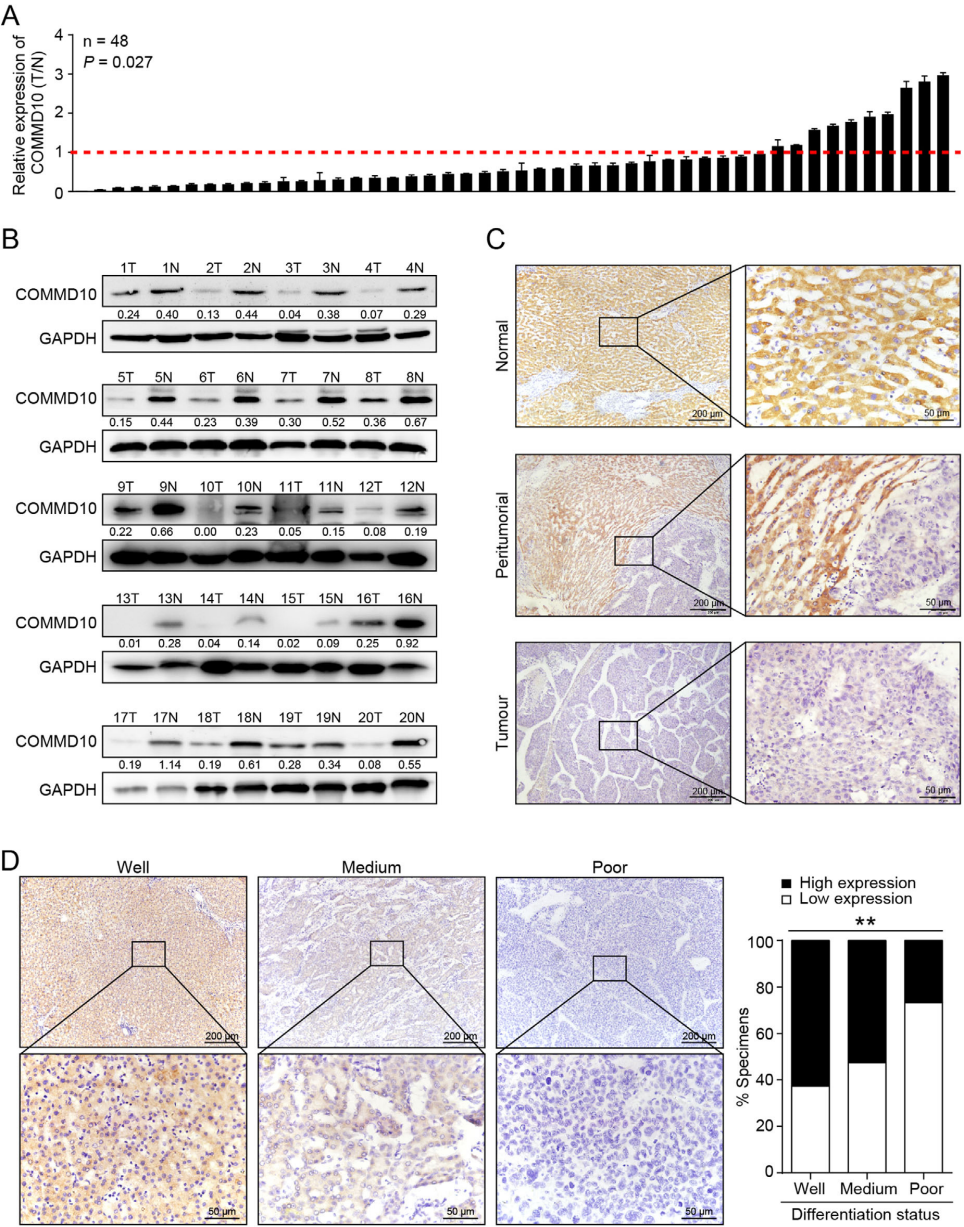
The association between COMMD10 expression and the clinical features in HCC. (A) Relative COMMD10 mRNA levels in 48 paired primary HCC tissues and matched normal liver tissues by real‐time PCR analysis. GAPDH mRNA was used as internal control. The data are shown from a single representative experiment of three repeats. (B) COMMD10 protein expression in 20 paired primary HCC tissues (T) and matched normal tissues (N); GAPDH was used as loading control. (C) Strong expression of COMMD10 protein in paraffin‐embedded normal, peritumorial liver tissues, and weak expression of COMMD10 in paraffin‐embedded HCC tissues by IHC staining analysis. (D) COMMD10 protein expression in well, medium, and poor differentiated paraffin‐embedded HCC tissues by IHC staining analysis in NF training cohort. Statistical quantification graph was shown in the right panel. Each bar represents the mean ± SD; *p < 0.05; **p < 0.01; ***p < 0.001

To explore the correlations between COMMD10 and clinicpathologic features in HCC, the expression of COMMD10 was measured in HCC samples. Real‐time PCR and western blots results showed that COMMD10 was significantly decreased at both mRNA (*p* = 0.027) and protein (*p* < 0.001) levels in the HCC tissues in comparison with that in normal liver tissues (Figures 5A, 5B, and S6). Moreover, IHC staining results showed that COMMD10 expression was remarkably reduced in tumor regions compared to that in healthy human liver (Figure 5C).

To further assess the clinical values of COMMD10, we evaluated the relationship between COMMD10 expression and clinicpathologic parameters in HCC patients from multi‐centered cohorts. COMMD10 expression was divided into low and high subgroup according to the optimal cut‐off value automatically generated by X‐tile software. A total of 48.1% (125 of 260), 44% (59 of 134), and 54.9% (67 of 122) tumors were scored as low COMMD10 expression in the Nanfang (NF) training, Nanfang (NF) internal and Zhujiang and Yuebei Hospital (ZY) external validation sets, respectively. Table 1 shows the clinical characteristics of the patients in the NF training cohort (*n* = 260), NF internal validation cohort (*n* = 134), and ZY external validation cohort (*n* = 122). Median follow‐up was 34.0 months (IQR 11.8‐54.3) for patients in the NF training cohort, 34.0 months (IQR 9.0‐45.0) for those in the NF internal validation cohort, and 38.5 months (IQR 14.8‐52.3) for those in the ZY external validation cohort. More than 80% of the patients were men, and most HCC was hepatitis B virus (HBV)‐related in the three sets. Most patients were diagnosed as stage 0 or A, which were considered as early stage according to BCLC stage. In addition, high expression of COMMD10 negatively correlated with tumor size while positively correlated with tumor differentiation in training cohort. Moreover, COMMD10 staining density decreased gradually accompanied with disease progression from well to poor differentiation in HCC specimens (*p* = 0.005, Figure 5D).

**TABLE 1:** Clinical characteristics of patients in the training, internal, and external validation cohorts

	NF training cohort (*n* = 260)				NF internal validation cohort (*n* = 134)				ZY external validation cohort (*n* = 122)			
	Patients	COMMD10			Patients	COMMD10			Patients	COMMD10		
Characteristic	Number	Low (%)	High (%)	*P* value	Number	Low (%)	High (%)	*P* value	Number	Low (%)	High (%)	*P* value
Age (years)				0.643				0.960				0.912
≤50	137	64 (46.7)	73 (53.3)		61	27 (44.3)	34 (55.7)		65	36 (55.4)	29 (44.6)	
>50	123	61 (49.6)	62 (50.4)		73	32 (43.8)	41 (56.2)		57	31 (54.4)	26 (46.6)	
Sex				0.157				0.018				0.664
Female	28	17 (60.7)	11 (39.3)		17	12 (70.6)	5 (29.4)		22	13 (59.1)	9 (40.7)	
Male	232	108 (46.6)	124 (53.4)		117	47 (40.2)	70 (59.8)		100	54 (54.0)	46 (46.0)	
Hepatitis				0.116				0.773				0.323
HBV	231	112 (48.5)	119 (51.5)		129	58 (45.0)	71 (55.0)		100	54 (54.0)	46(46.0)	
HCV	5	4 (80.0)	1 (20.0)		1	0(0)	1 (100.0)		3	1 (33.3)	2 (66.7)	
Unknown	24	9 (37.5)	15 (62.5)		4	1 (25.0)	3 (75.0)		19	12 (63.2)	7 (36.)	
Tumor size (cm)				0.018				0.078				0.998
≤5	130	53 (40.8)	77 (59.2)		75	28 (37.3)	47 (62.7)		51	28 (54.9)	23 (45.1)	
>5	130	72 (55.4)	58 (44.6)		59	31 (52.5)	28 (47.5)		71	39 (54.9)	32 (45.0)	
Tumor number				0.148				0.670				0.718
1	217	100 (46.1)	117 (53.9)		121	54 (44.6)	67 (55.4)		110	61 (55.5)	49 (4.5)	
≥2	43	25 (58.1)	18 (41.9)		13	5 (38.5)	8 (61.5)		12	6 (50.0)	6 (50.0)	
Tumor embolus				0.677				0.471				1.000
Yes	29	15 (51.7)	14 (48.3)		9	5 (55.6)	4 (44.4)		11	6 (54.5)	5 (45.5)	
No	231	110 (47.6)	121 (52.4)		125	54 (43.2)	71 (56.8)		110	60 (54.5)	50 (45.5)	
Unknown	0	0	0		0	0	0		1	1 (100)	0(0)	
Tumor differentiation				0.002				0.002				0.289
Well	59	22 (37.3)	37 (62.7)		32	10 (31.3)	22 (68.8)		7	4 (57.1)	3 (42.9)	
Medium	171	81 (47.4)	90 (52.6)		88	37 (42.0)	51 (58.0)		98	51 (52.0)	47 (48.0)	
Poor	30	22 (73.3)	8 (26.7)		14	12 (85.7)	2 (14.3)		17	12 (70.6)	5 (29.4)	
Cirrhosis				0.907				0.917				0.828
Yes	186	89 (47.8)	97 (52.2)		96	42 (43.8)	54 (56.3)		59	33 (55.9)	26 (44.1)	
No	84	36 (48.6)	38 (51.4)		38	17 (44.7)	21 (55.3)		63	34 (54.0)	29 (46.0)	
AFP (μg/L)				0.074				0.010				0.855
<400	150	65 (43.3)	85 (56.7)		100	38 (38.0)	62 (62.0)		68	38 (55.9)	30 (44.1)	
≥400	110	60 (54.5)	50 (45.5)		33	21 (63.6)	12 (36.4)		48	26 (54.2)	22 (45.8)	
Unknown	0	0()	0()		1	0(0)	1 (100)		6	3 (50.0)	3 (50.0)	
TBIL (μmol/L)				0.901				0.292				0.085
≤17.1	180	87 (48.3)	93 (51.7)		96	45 (46.9)	51 (53.1)		83	50 (60.2)	33 (39.8)	
>17.1	80	38 (47.5)	42 (52.5)		38	14 (36.8)	24 (63.2)		39	17 (43.6)	22 (56.4)	
ALB (g/L)				0.084				0.346				0.162
≤35	49	29 (59.2)	20 (40.8)		29	15 (51.7)	14 (48.3)		17	12 (70.6)	5 (29.4)	
>35	211	96 (45.5)	115 (54.5)		105	44 (41.9)	61 (58.1)		105	55 (52.4)	50 (47.6)	
BCLC stage				0.218				0.981				0.825
0 and A	204	94 (46.1)	110 (53.9)		118	52 (44.1)	66 (55.9)		98	53 (54.1)	45 (45.9)	
B and C	56	31 (55.4)	25 (44.6)		16	7 (43.8)	9 (56.3)		24	14 (58.3)	10 (41.7)	
Postoperative treatment				0.002				0.005				0.009
TACE	125	62 (49.6)	63 (50.4)		72	33 (45.8)	39 (54.2)		49	19 (38.8)	30 (61.2)	
RFA	18	5 (27.8)	13 (72.2)		10	1 (10.0)	9 (90.0)		7	3 (42.9)	4 (57.1)	
Target	13	5 (38.5)	8 (61.5)		8	3 (37.5)	5 (62.5)		0	0()	0()	
Reoperation	25	10 (40.0)	15 (60.0)		9	4 (44.4)	5 (55.6)		7	5 (71.4)	2 (28.6)	
Biotherapy	7	3 (42.9)	4 (57.1)		9	3 (33.3)	6 (66.7)		0	0()	0()	
Chemotherapy	14	9 (64.3)	5 (35.7)		9	2 (22.2)	7 (77.8)		4	2 (50.0)	2 (50.0)	

Abbreviations: AFP, α‐fetoprotein; ALB, Albumin; BCLC stage, Barcelona Clinic Liver Cancer stage; HBV, hepatitis B virus; HCV, hepatitis C virus; TBIL, total bilirubin.

### 3.6 COMMD10 is a predictor of stratification and prognosis in HCC

**FIGURE 6:**
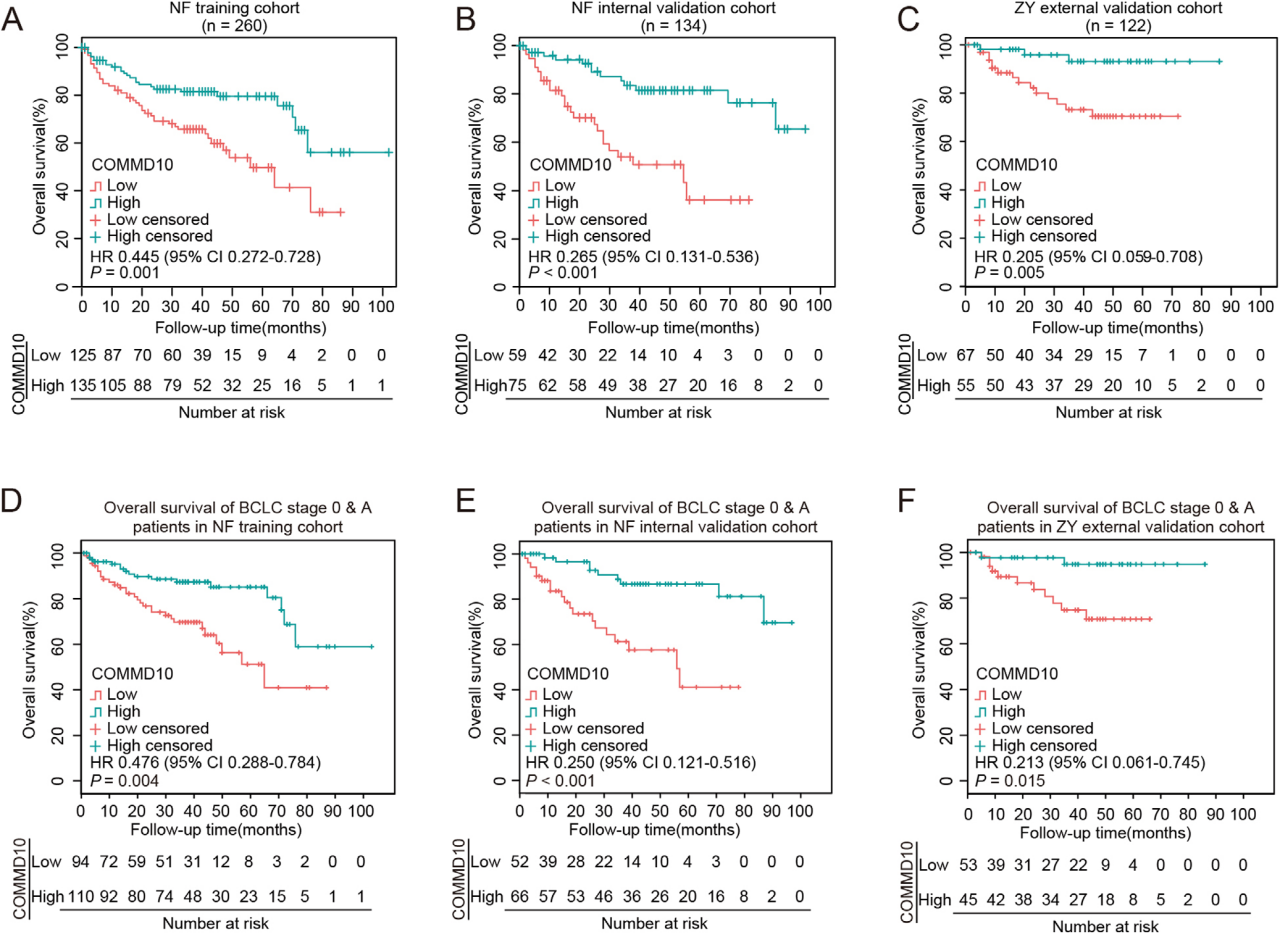
COMMD10 is a predictor of stratification and prognosis in HCC. (A‐C) Prediction of HCC patients’ OS based on COMMD10 expression in NF training cohort (A), NF internal validation cohort (B), and ZY external validation cohort (C) using Kaplan‐Meier survival analysis. (D‐F) Prediction of early HCC (BCLC stage 0 and A) patients’ OS based on COMMD10 expression in NF training cohort (D), NF internal validation cohort (E), and ZY external validation cohort (F) using Kaplan‐Meier survival analysis

To investigate whether COMMD10 expression was an independent prognostic predictor of OS, Kaplan–Meier survival analysis was applied to compare OS of HCC patients according to COMMD10 expression. Patients with low COMMD10 expression had a significantly poorer OS than those with a high COMMD10 expression in the NF training, NF internal, and ZY external validation cohorts (*p* = 0.001, *p* < 0.001, and *p* = 0.005, respectively; Figures 6A–6C). The median of 1‐, 2‐, 3‐, and 5‐year OS in three cohorts is shown in Table S2. We further examined whether COMMD10 expression could stratify patients with early‐stage (stage 0 and A) and late‐stage (stage B and C) according to BCLC stage. When the analysis was limited to early‐stage HCC, patients in three cohorts could be significantly stratified by COMMD10 expression (*p *= 0.001, *p* < 0.001, and *p* = 0.019 respectively; Figures 6D–6F). However, the COMMD10 expression was not predictive in late‐stage HCC in our three sets (*p* = 0.774, *p* = 0.154, and *p* = 0.486, respectively; Figures S7A‐S7C). The above findings suggest that COMMD10 is a predictor of stratification and prognosis in HCC.

### 3.7 Extension of the BCLC staging prognostic model with COMMD10 expression for HCC patients

**FIGURE 7:**
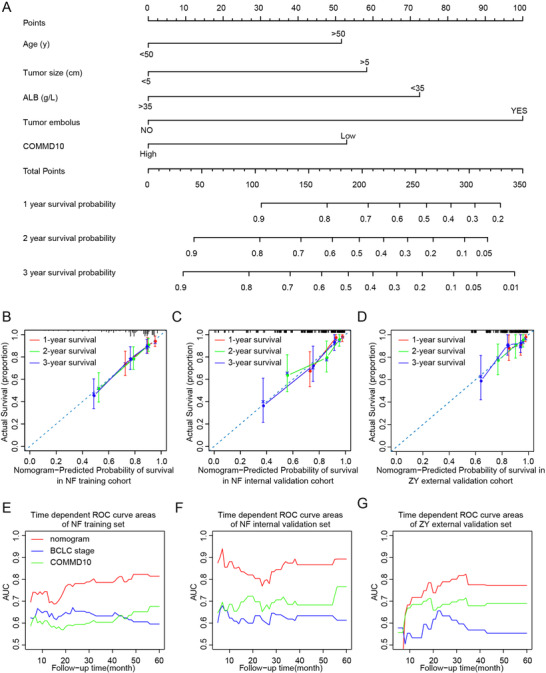
Development and evaluation of COMMD10‐based nomogram in HCC cohorts. (A) Nomogram, including patient age, ALB (g/L), tumor size (cm), embolus, and COMMD10 expression for 1‐, 2‐, 3‐year overall survival in HCC patients. The nomogram allows the user to obtain the probability of 1‐, 2‐, 3‐year overall survival corresponding to a patient's combination of covariates. (B‐D) The calibration curve of nomogram for predicting overall survival at 1‐, 2‐, 3‐year in NF training cohort (B), NF validation cohort (C) and ZY external validation cohort (D). Actual overall survival is plotted on the y‐axis; nomogram predicted probability of overall survival is plotted on the x‐axis. (E‐G) Time dependent ROC curve areas of nomogram, BCLC stage system, and COMMD10 expression in NF training cohort (E), NF internal validation cohort (F), and ZY external validation cohort (G)

To investigate whether incorporation of the COMMD10 expression into the clinicpathologic variables of BCLC system would improve its predictive accuracy, we firstly performed univariate analysis and found various prognosis factors for OS in the NF training cohort, such as age, tumor size, albumin (ALB), tumor embolus, tumor differentiation, and COMMD10 (Table S3). Multivariate Cox regression analysis revealed that COMMD10, age, tumor size, tumor embolus, and ALB remained strong and independent prognostic factors for OS in the NF training cohort (Table S4). Then we generate a composite prognostic nomogram including COMMD10, age, tumor size, tumor embolus, and ALB level to predict 1‐, 2‐, and 3‐year OS in the NF training cohort (Figure 7A). Each factor in the nomogram was assigned a number of weighted points, and the total points of each patient were relevant to a specificity prediction of 1‐, 2‐, and 3‐year OS. The bootstrapped calibration plots of 1‐, 2‐, and 3‐year OS showed that nomogram prediction was consistent with the actual observation in three cohorts (Figures 7B–7D, respectively).

Moreover, the predictive power for OS of HCC between nomogram, BCLC stage, and COMMD10 was compared. As shown in Table S5, the C‐index of nomogram for OS prediction was higher than that of BCLC stage alone (*p* = 0.013, *p* < 0.001, *p* = 0.007, respectively) and COMMD10 alone (*p* < 0.001, *p* < 0.001, *p* = 0.216, respectively) in three cohorts. The time dependent ROC curve areas to compare the sensitivity and specificity of the nomogram showed better prognostic value of the OS compared to BCLC staging system alone and COMMD10 alone in three cohorts (Figures 7E–7G, respectively). These results suggest that COMMD10 provides independent and auxiliary prognostic information in HCC patients and that combination of this information with the BCLC staging can enhance prognostic stratification.

## 4 DISCUSSION

HCC is the fourth leading reason for cancer mortality worldwide.^21^ To date, there is limited knowledge of dysregulation of cellular proliferation and apoptosis that contribute to the malignant phenotype in HCC.^22^ COMMD10 is initially identified as a suppressor gene in the pathogenesis of HCC in our observations.^5^ However, the underlying mechanisms and clinical values of COMMD10 in suppressing HCC progression remain unknown.

COMMD10, a member of the Copper Metabolism MURR1 Domain‐containing protein family, is evolutionary conserved in vertebrate^23^ and involved in multiple biological processes, such as staphylococcus clearance,^10^ inflammation inhibition,^11^ and Na^+^ transport.^12^ The transcription factor NF‐κB has been shown to be activated in HCC,^7^ which displays aggressive pathologic features and has poor treatment outcomes.^24^ In our study, we found that overexpressing COMMD10 dramatically reduced, whereas silencing COMMD10 promoted the NF‐κB luciferase activity. The effects of COMMD10 silence on the proliferation in HCC cells in vitro and in vivo were reversed by NF‐κB inhibitor (IκBα‐mut), suggesting that COMMD10 suppresses proliferation via NF‐κB pathway. Furthermore, we found that COMMD10 suppressed NF‐κB signaling pathways by interacting and inhibiting p65 translocation to the nucleus in HCC. Unlike previous study which identified that COMMD10 could associate more broadly with NF‐κB subunits in HEK293T cells,^23^ we detect no binding affinity between COMMD10 and other endogenous NF‐κB subunits except p65 in HepG2 cells, which may indicate that the mechanism by which COMMD10 regulates NF‐κB is different across cell types. Although all 10 COMMD proteins interact with NF‐κB, the COMMD proteins inhibit NF‐κB in different way.^9^ COMMD1 interacts with IκBα to inhibit IκBα proteasomal degradation in the cytosol and promote p65 nucleolar retention and proteasomal degradation.^25^ Contrary to COMMD1, no combination was found between COMMD6 and IκBα.^9^ Interestingly, others reported that the COMMD domain was required for the interaction between COMMD members and NF‐κB,^26^, ^27^ our results show that N‐terminal domain of COMMD10 suppresses p65 nuclear translocation through binding to the RHD of p65. Since the N‐terminal region varies, this region might confer specific functions on COMMD10, which needs to be further investigated.

Previous studies have reported the role of NF‐κB signaling in modulating apoptosis.^20^ Some reports suggested a pro‐apoptotic role of NF‐κB in serum starvation of HEK293 cells,^28^ fibroblasts from ataxia telangiectasia patients,^29^ ultraviolet light, and daunorubicin/doxorubicin models.^30^ Many other reports, however, indicated NF‐κB has an anti‐apoptotic effect when exposed to radiation, cytotoxic drug, and inflammatory cytokines.^31^, ^32^ In our study, we found COMMD10 overexpression enhanced radiation and cis‐platinum‐ induced apoptosis by modulating Bcl‐2/Bax/caspase‐9/3 pathway in HCC cells. The intrinsic apoptosis pathway is principally regulated by the Bcl‐2 family, which contains NF‐κB binding sites.^33^ Some of Bcl‐2 family members (such as Bax, Bad, Bak, or Bid) promote apoptosis, while other members (such as Bcl‐2, Bax‐XL, Bcl‐XS, and Bcl‐XL) inhibit the role in the regulation of apoptosis.^34^, ^35^ The response of cells to various stimulation was determined by the proportion of pro‐ and anti‐apoptotic Bcl‐2 proteins. In our study, we found that overexpressing COMMD10 significantly reduced the expression of Bcl‐2 (anti‐apoptotic) and enhanced the Bax (pro‐apoptotic) level. We also found that extrinsic apoptosis pathway inducer cleaved caspase‐8 was elevated in COMMD10‐overexpressing HCC cells, but was downregulated in COMMD10‐silenced HCC cells. However, cleaved caspase‐8 expression was unchanged when NF‐κB activity was inhibited by IκBα‐mut, indicating that the apoptosis pathway involving caspase8 might not be a downstream target of the COMMD10/NF‐κB pathway. Therefore, our findings validate that COMMD10/NF‐κB axis promoted intrinsic apoptosis by modulating Bcl‐2/Bax/caspase‐9/3 pathway.

Accurate prognostic assessment is of great clinical value in guiding the clinical management and selecting appropriate therapy. However, the current HCC patients determined by the BCLC stage, which is recognized as the best guideline for HCC management, show different prognosis although in the same disease stage.^36^, ^37^ This is most likely because this system does not take tumor heterogeneity into account. Despite that several nomograms have been established to improve the accuracy in predicting survival outcomes of HCC patients,^38^, ^39^, ^40^ the predictive factors in these nomograms are confined to characteristics of patients and their tumors. However, biological heterogeneity remains the main factor causing the difference in prognosis. Some authors have proposed to refine the prognosis scoring by introducing tumor molecular biomarkers, such as mRNA‐based genes, and alternative genomic sources (e.g., microRNAs or epigenomics).^41^, ^42^ Thus, novel molecular biomarkers for identifying tumor heterogeneity and accurately predicting clinical outcomes of HCC are urgently required.

In this study, we verified that differential expression of COMMD10 caused difference in HCC proliferation and survival rate, making it a potential molecular biomarker for distinguishing HCC heterogeneity. Then we describe the construction and validation of a composite prognostic nomogram combining COMMD10 and clinic‐pathological variables of BCLC system to predict 1‐, 2‐, and 3‐year OS for HCC patients from multi‐centered cohorts, which yielded better predictive accuracy than the BCLC staging system. What is more, the external validation of GSE14520 used microarray‐based data generated from 219 fresh HCC tissues also revealed that patients with low COMMD10 level showed shorter OS (*p* = 0.048, Figure S8), which further supports the prediction values of COMMD10 for HCC patients.

Additionally, we found that COMMD10 level categorized patients into high‐risk and low‐risk groups of early‐stage (BCLC stage 0 and A) HCC patients who had significantly different OS. In HCC, the 5‐year survival rate achieves up to 70% if patients are diagnosed at an early stage.^43^ Despite being diagnosed with early stage, approximately 60% at 5 years of HCC patients eventually experienced recurrence.^44^ These imply that defining molecular subgroups by COMMD10 may identify patients who are more likely to relapse and require more individual therapy, and ultimately improve their survival outcomes. However, the COMMD10 expression was difficult to quantify risk in late‐stage HCC in our three sets, the most likely reason is the diversity of comorbidities and the insufficient sample size of advanced HCC patients.

There were some limitations in this study. First, our data were available in China, and more than 80% of HCC was HBV‐related in the three cohorts. However, the distribution of pathological subtypes and clinical features might be different in other countries. COMMD10‐based nomogram requires further validation in prospective studies and multicenter clinical trials. Second, since various causes of HCC, such as HBV infection, hepatitis C virus infection, alcoholic liver disease, non‐alcoholic steatohepatitis, environmental and dietary carcinogens factors could contribute to the development and progression of HCC, the mechanism of COMMD10 in regulating different types of HCC might make a great difference. Further work is warranted to obtain precise mechanism of COMMD10 in the occurrence and progression of HCC.

## CONFLICT OF INTEREST

The authors declare that they have no competing interests.

## AUTHOR CONTRIBUTIONS

Professor Jian Guan conceived the idea, wrote and proofread the manuscript. Professor Longhua Chen and Li Liang analyzed data and proofread the manuscript. Mi Yang wrote and proofread the manuscript. Mi Yang, Xixi Wu, LuLi, Shaoqun Li, Suming Pan, Richang Du, Xiaoqing Wang, Min Chen, Nanjie Xiao, Xiaohui Zhu, and Guoyang He carried out in vitro experiments. Mi Yang, Nan Li, Mengyuan Mao, Longshan Zhang, Weiqiang Huang, Hua Pan, and Lan Deng gave assistance in collecting tissue samples or performing animal experiments and carrying out the statistical analysis. All authors read and approved the final manuscript.

## ETHICS APPROVAL AND CONSENT TO PARTICIPATE

Informed consent was obtained to bank HCC specimen for research purposes. The collection of HCC specimen for research purpose was approved by the Ethics Committee of the Nanfang Hospital. All animal experiments were conducted in accordance with the principles and procedures approved by the Committee on the Ethics of Animal Experiments of Southern Medical University.

## CONSENT FOR PUBLICATION

All authors reached an agreement to publish the study in this journal.

## Supporting information

SUPPORTING INFORMATION

SUPPORTING INFORMATION

SUPPORTING INFORMATION

## References

[1] Sung H , Ferlay J , Siegel RL , et al. Global cancer statistics 2020: GLOBOCAN estimates of incidence and mortality worldwide for 36 cancers in 185 countries. CA Cancer J Clin. 2021. 10.3322/caac.21660. 33538338

[2] Allemani C , Matsuda T , Di Carlo V , et al. Global surveillance of trends in cancer survival 2000–14 (CONCORD‐3): analysis of individual records for 37 513 025 patients diagnosed with one of 18 cancers from 322 population‐based registries in 71 countries. Lancet. 2018;391:1023–1075. 29395269 10.1016/S0140-6736(17)33326-3 PMC5879496

[3] Liang L , Guan J , Zeng Y , et al. Down‐regulation of formin‐like 2 predicts poor prognosis in hepatocellular carcinoma. Hum Pathol. 2011;42:1603–1612. 21496865 10.1016/j.humpath.2010.08.025

[4] Yang SS , Li XM , Yang M , et al. FMNL2 destabilises COMMD10 to activate NF‐kappaB pathway in invasion and metastasis of colorectal cancer. Br J Cancer. 2017;117:1164–1175. 28817833 10.1038/bjc.2017.260 PMC5674093

[5] Fan Y , Zhang L , Sun Y , et al. Expression profile and bioinformatics analysis of COMMD10 in BALB/C mice and human. Cancer Gene Ther. 2019;27:216–225. 30787448 10.1038/s41417-019-0087-9

[6] Freimuth J , Bangen JM , Lambertz D , et al. Loss of caspase‐8 in hepatocytes accelerates the onset of liver regeneration in mice through premature nuclear factor kappa B activation. Hepatology. 2013;58:1779–1789. 23728913 10.1002/hep.26538

[7] Dai T , Zhang D , Cai M , et al. Golgi phosphoprotein 3 (GOLPH3) promotes hepatocellular carcinoma cell aggressiveness by activating the NF‐kappaB pathway. J Pathol. 2015;235:490–501. 25385148 10.1002/path.4479

[8] Vainer GW , Pikarsky E , Ben‐Neriah Y . Contradictory functions of NF‐kappaB in liver physiology and cancer. Cancer Lett. 2008;267:182–188. 18479806 10.1016/j.canlet.2008.03.016

[9] de Bie P , van de Sluis B , Burstein E , et al. Characterization of COMMD protein‐protein interactions in NF‐kappaB signalling. Biochem J. 2006;398:63–71. 16573520 10.1042/BJ20051664 PMC1525016

[10] Ben Shlomo S , Mouhadeb O , Cohen K , Varol C , Gluck N . COMMD10‐guided phagolysosomal maturation promotes clearance of staphylococcus aureus in macrophages. iScience. 2019;14:147–163. 30959277 10.1016/j.isci.2019.03.024 PMC6453835

[11] Mouhadeb O , Ben Shlomo S , Cohen K , et al. Impaired COMMD10‐mediated regulation of Ly6C(hi) monocyte‐driven inflammation disrupts gut barrier function. Front Immunol. 2018;9:2623. 30487795 10.3389/fimmu.2018.02623 PMC6246736

[12] Ware AW , Cheung TT , Rasulov S , Burstein E , McDonald FJ . Epithelial Na(+) channel: reciprocal control by COMMD10 and Nedd4‐2. Front Physiol. 2018;9:793. 29997525 10.3389/fphys.2018.00793 PMC6028986

[13] Aslibekyan S , Kabagambe EK , Irvin MR , et al. A genome‐wide association study of inflammatory biomarker changes in response to fenofibrate treatment in the genetics of lipid lowering drug and diet network. Pharmacogenet Genomics. 2012;22:191–197. 22228203 10.1097/FPC.0b013e32834fdd41 PMC3275691

[14] Smolonska J , Koppelman GH , Wijmenga C , et al. Common genes underlying asthma and COPD? Genome‐wide analysis on the Dutch hypothesis. Eur Respir J. 2014;44:860–872. 24993907 10.1183/09031936.00001914 PMC4217133

[15] Nome T , Thomassen GO , Bruun J , et al. Common fusion transcripts identified in colorectal cancer cell lines by high‐throughput RNA sequencing. Transl Oncol. 2013;6:546–553. 24151535 10.1593/tlo.13457 PMC3799197

[16] Camp RL , Dolled‐Filhart M , Rimm DL . X‐tile: a new bio‐informatics tool for biomarker assessment and outcome‐based cut‐point optimization. Clin Cancer Res. 2004;10:7252–7259. 15534099 10.1158/1078-0432.CCR-04-0713

[17] Verslype C , Rosmorduc O , Rougier P , Group EGW . Hepatocellular carcinoma: ESMO‐ESDO clinical practice guidelines for diagnosis, treatment and follow‐up. Ann Oncol. 2012;23(Suppl 7):vii41–vii48. 22997453 10.1093/annonc/mds225

[18] Harrell FE Jr , Lee KL , Mark DB . Multivariable prognostic models: issues in developing models, evaluating assumptions and adequacy, and measuring and reducing errors. Stat Med. 1996;15:361–387. 8668867 10.1002/(SICI)1097-0258(19960229)15:4<361::AID-SIM168>3.0.CO;2-4

[19] Lecoq L , Raiola L , Chabot PR , et al. Structural characterization of interactions between transactivation domain 1 of the p65 subunit of NF‐kappaB and transcription regulatory factors. Nucleic Acids Res. 2017;45:5564–5576. 28334776 10.1093/nar/gkx146 PMC5435986

[20] Pan X , Arumugam T , Yamamoto T , et al. Nuclear factor‐kappaB p65/relA silencing induces apoptosis and increases gemcitabine effectiveness in a subset of pancreatic cancer cells. Clin Cancer Res. 2008;14:8143‐8151. 19088029 10.1158/1078-0432.CCR-08-1539 PMC4403242

[21] Bray F , Ferlay J , Soerjomataram I , Siegel RL , Torre LA , Jemal A . Global cancer statistics 2018: GLOBOCAN estimates of incidence and mortality worldwide for 36 cancers in 185 countries. CA Cancer J Clin. 2018;68:394–424. 30207593 10.3322/caac.21492

[22] Bimonte S , Albino V , Barbieri A , et al. Dissecting the roles of thymoquinone on the prevention and the treatment of hepatocellular carcinoma: an overview on the current state of knowledge. Infect Agent Cancer. 2019;14:10. 31015860 10.1186/s13027-019-0226-9 PMC6469080

[23] Burstein E , Hoberg JE , Wilkinson AS , et al. COMMD proteins, a novel family of structural and functional homologs of MURR1. J Biol Chem. 2005;280:22222–22232. 15799966 10.1074/jbc.M501928200

[24] Arsura M , Cavin LG . Nuclear factor‐kappaB and liver carcinogenesis. Cancer Lett. 2005;229:157–169. 16125305 10.1016/j.canlet.2005.07.008

[25] Ganesh L , Burstein E , Guha‐Niyogi A , et al. The gene product Murr1 restricts HIV‐1 replication in resting CD4+ lymphocytes. Nature. 2003;426:853–857. 14685242 10.1038/nature02171

[26] Maine GN , Burstein E . COMMD proteins: COMMing to the scene. Cell Mol Life Sci. 2007;64:1997–2005. 17497243 10.1007/s00018-007-7078-y PMC2938186

[27] Sommerhalter M , Zhang Y , Rosenzweig AC . Solution structure of the COMMD1 N‐terminal domain. J Mol Biol. 2007;365:715–721. 17097678 10.1016/j.jmb.2006.10.030 PMC2706016

[28] Grimm S , Baeuerle PA . Failure of the splicing variant p65 delta of the NF‐kappa B subunit p65 to transform fibroblasts. Oncogene. 1994;9:2391–2398. 8036023

[29] Jung M , Zhang Y , Lee S , Dritschilo A . Correction of radiation sensitivity in ataxia telangiectasia cells by a truncated I kappa B‐alpha. Science. 1995;268:1619–1621. 7777860 10.1126/science.7777860

[30] Campbell KJ , Rocha S , Perkins ND . Active repression of antiapoptotic gene expression by RelA(p65) NF‐kappa B. Mol Cell. 2004;13:853–865. 15053878 10.1016/s1097-2765(04)00131-5

[31] Barkett M , Gilmore TD . Control of apoptosis by Rel/NF‐kappaB transcription factors. Oncogene. 1999;18:6910–6924. 10602466 10.1038/sj.onc.1203238

[32] Baldwin AS, Jr . The NF‐kappa B and I kappa B proteins: new discoveries and insights. Annu Rev Immunol. 1996;14:649–683. 8717528 10.1146/annurev.immunol.14.1.649

[33] Glasgow JN , Wood T , Perez‐Polo JR . Identification and characterization of nuclear factor kappaB binding sites in the murine bcl‐x promoter. J Neurochem. 2000;75:1377–1389. 10987817 10.1046/j.1471-4159.2000.0751377.x

[34] Jurgensmeier JM , Xie Z , Deveraux Q , Ellerby L , Bredesen D , Reed JC . Bax directly induces release of cytochrome c from isolated mitochondria. Proc Natl Acad Sci U S A. 1998;95:4997–5002. 9560217 10.1073/pnas.95.9.4997 PMC20202

[35] Youle RJ , Strasser A . The BCL‐2 protein family: opposing activities that mediate cell death. Nat Rev Mol Cell Biol. 2008;9:47–59. 18097445 10.1038/nrm2308

[36] Forner A , Llovet JM , Bruix J . Hepatocellular carcinoma. Lancet. 2012;379:1245–1255. 22353262 10.1016/S0140-6736(11)61347-0 PMC13537732

[37] Kulik L , El‐Serag HB . Epidemiology and management of hepatocellular carcinoma. Gastroenterology. 2019;156:477–491. 30367835 10.1053/j.gastro.2018.08.065 PMC6340716

[38] Li Y , Xia Y , Li J , et al. Prognostic nomograms for pre‐ and postoperative predictions of long‐term survival for patients who underwent liver resection for huge hepatocellular carcinoma. J Am Coll Surg. 2015;221:962–974. 26382973 10.1016/j.jamcollsurg.2015.08.003

[39] Shim JH , Jun MJ , Han S , et al. Prognostic nomograms for prediction of recurrence and survival after curative liver resection for hepatocellular carcinoma. Ann Surg. 2015;261:939–946. 24950276 10.1097/SLA.0000000000000747

[40] Feng LH , Dong H , Lau WY , et al. Novel microvascular invasion‐based prognostic nomograms to predict survival outcomes in patients after R0 resection for hepatocellular carcinoma. J Cancer Res Clin Oncol. 2017;143:293–303. 27743138 10.1007/s00432-016-2286-1 PMC11819416

[41] Forner A , Bruix J . Biomarkers for early diagnosis of hepatocellular carcinoma. Lancet Oncol. 2012;13:750–751. 22738800 10.1016/S1470-2045(12)70271-1

[42] Villanueva A , Hoshida Y , Toffanin S , et al. New strategies in hepatocellular carcinoma: genomic prognostic markers. Clin Cancer Res. 2010;16:4688–4694. 20713493 10.1158/1078-0432.CCR-09-1811 PMC3395071

[43] Llovet JM , Zucman‐Rossi J , Pikarsky E , et al. Hepatocellular carcinoma. Nat Rev Dis Primers. 2016;2:16018. 27158749 10.1038/nrdp.2016.18

[44] Tabrizian P , Jibara G , Shrager B , Schwartz M , Roayaie S . Recurrence of hepatocellular cancer after resection: patterns, treatments, and prognosis. Ann Surg. 2015;261:947–955. 25010665 10.1097/SLA.0000000000000710

