# Supplementary material for: COMMD10 inhibits tumor progression and induces apoptosis by blocking NF‐κB signal and values up BCLC staging in predicting overall survival in hepatocellular carcinoma
Source: Clin Transl Med. 2021 May 4;11(5):e403. doi: 10.1002/ctm2.403 (PMC8093973; doi:10.1002/ctm2.403)
Supplement: Supplementary file 1 — SUPPORTING INFORMATION [file CTM2-11-e403-s002.docx]

**Supplementary Materials and Methods**

**Tissue specimens**

Paraffin-embedded HCC samples were collected from 516 patients who underwent HCC resection at the Department of Hepatobiliary Surgery in Nanfang, Zhujiang and Yuebei hospital. Fresh HCC samples and matched adjacent non-tumor tissues were collected immediately after resection, snap-frozen in liquid nitrogen, and then stored at -80 °C until needed. Prior patient’s consent and Institutional Research Ethics Committee approval were obtained for the use of these clinical materials for research purposes.

**Procedures**

All patients were graded according to BCLC stage and diagnosed with HCC after surgery based on pathologic analysis. A detailed history, findings of a complete physical examination, and laboratory test and imaging examination results were retrieved from patients’ medical records. Laboratory tests, including liver function tests, assays of AFP and carbohydrate antigen 19-9 levels, and blood coagulation tests, were performed up to 7 days before surgery. Imaging examinations, including magnetic resonance imaging, computed tomography scanning, and ultrasonography, were performed up to 14 days before surgery. This study was approved by the Ethics Committee of Nanfang Hospital of Southern Medical University, and all participants provided written informed consent prior to surgery. It also conforms to the provisions of the Declaration of Helsinki.

**Quantitative RT-PCR**

Total RNA was extracted from cells using RNAiso Plus (TaKaRa Biotechnology, Dalian, China) following the manufacturer’s protocol. The complementary DNA (cDNA) library was synthesized using the Prime Script RT reagent kit (TaKaRa Biotechnology, Dalian, China). The PCR primers for COMMD10 were as follows: forward 5′-ATAGTGTTGCTTTCTTGCCTGTC-3′ and reverse 5′-ACCAAAGCTC TGTATTCTACTAAGG-3′. The PCR primers for GADPH were as follows: forward 5′-CCATCAATGACCCCTTCATTGACC-3′ and reverse 5′-GAAGGCCATGCCAGTGAGCTTCC-3′. RT-PCR for messenger RNA (mRNA) were performed using SYBRP remix Ex Taq II (TaKaRa Biotechnology, Dalian, China), and the RT-PCR products were measured with an ABI 7500 Sequence Detection System (Perkin Elmer/Applied Biosystems Group, Foster City, CA, USA). GADPH was used as the internal control. The mRNA expression levels were quantified using the delta-delta Ct method.

**Plasmids and siRNA transfection，lentivirus shRNA gene infection**

For overexpression of COMMD10, COMMD10 plasmid (GeneCopoeia, USA) was transfected into SMMC-7721 and HepG2 cells. The pCDEF-flag-IκBα-mut plasmid with mutation at S32 and S36 was purchased from GeneCopoeia (GeneCopoeia, USA). For depletion of COMMD10, human siRNA1, siRNA2 and siRNA3 sequences (sense, 5’-GCA AUA GAU ACA GGA AGAU dTdT-3’; sense, 5’-GCA GCA AUU AGA GAA CAAU dTdT-3’; sense, 5’ GGC AGC UUA ACC UUC AGAU dTdT-3’) were used to transfect HepG2 and QGY-7703 cells, and a scramble siRNA (5’-AAT CGC ATA GCG TAT GCC GTT-3’), which has no homology with the mammalian mRNA sequences, was used as control. Cells were transfected with 4 μg of plasmids or 100 nM siRNAs using Lipofectamine^®^2000 according to the instructions (Invitrogen, Carlsbad, CA, USA). HepG2 cells were plated in 24-well plates (2×10^4^ cells/well) and infected with COMMD10 shRNA lentivirus (Shanghai Genechem, Shanghai, China) to construct HepG2/shCOMMD10 cells. HepG2/shCOMMD10 cells were infected with IκBα lentivirus (Shanghai Genechem, Shanghai, China) to construct HepG2/shCOMMD10/IκBα stable cells. Briefly, the lentiviruses were diluted in 0.5 ml (10^8^ TU/ml) enhanced infection solution (ENi.S.) containing polybrene (8 mg/ml) and added to the cells for a 12-h incubation at 37°C, and then replaced with fresh 1640 medium and the cells were cultured for next 48 h, followed by selection for 10 days with 0.5 mg/ml puromycin. Western blot analysis was performed to detect COMMD10 expression.

**Western blot**

Western blot analysis was performed according to standard methods as described previously[[1](#_ENREF_1)]. Antibodies used for western blot analysis were as follows: apoptosis antibody sampler kit and NF-κB family member kit (Cell Signaling Technology, Inc., Danvers, MA, USA); anti-COMMD10, anti-Bcl2, anti-Bax antibodies (Abcam, Cambridge, MA, UK); anti-XIAP and anti-cIAP-1 (Cell Signaling Technology, Inc., Danvers, MA, USA); anti-GAPDH antibody (Proteintech Group, Rosemont, IL, USA) as a loading control.

**Clone formation**

HepG2/Mock, HepG2/COMMD10, HepG2/Vector and HepG2/siCOMMD10 cells were added to different wells of a 6-well culture plate at a final density of 1 × 10^2^ cells. After incubation for 2 weeks at 37°C, the plated cells were washed twice with PBS and stained with Giemsa solution. Colonies (≥50 cells as a clone) were counted under a dissecting microscope (plate clone formation efficiency = [number of colonies/number of cells inoculated] × 100%). All experiments were independently repeated at least three times.

**Cell proliferation assay**

COMMD10 deletion QGY-7703, HepG2 cells, and COMMD10 overexpression SMMC-7721, HepG2 cells were seeded into 96-well plates. The number of viable cells was determined by cell counting kit-8 (CCK-8) (Dojindo Molecular Technologies, Kumamoto, Japan) for 7 days. Briefly, 10μL CCK-8 solution was added, and absorbance at 450 nm was measured after 2 h of incubation at 37°C. Each cell group was plated in 3 duplicate wells.

**Immunofluorescence assay**

HepG2/COMMD10, HepG2/siCOMMD10 and corresponding control cells were plated on polylysine-treated slides overnight at 37°C. The cells were fixed in 4% paraformaldehyde for 10 min, blocked with phosphate-buffered saline (PBS) buffer containing 5% bovine serum albumin (BSA), and then incubated with rabbit anti-COMMD10 (1:100, Abcam), mouse anti-p65 (1:100, CST) at 4°C overnight, followed by incubation with fluorescein isothiocyanate (FITC)-conjugated secondary antibody (1:100 goat anti-rabbit immunoglobulin G [IgG], Santa Cruz Biotech) and the nuclear counterstain diaminophenylindole (DAPI). After rinsing, the cells were analyzed using immunofluorescence microscopy.

**Xenograft tumor model**

Male BALB/c nude mice (4–6 weeks old, 18–20 g) were purchased from the Guangdong Medical Laboratory Animal Center (Guangdong, China) and treated according to the guidelines established by the National Institutes of Health Guide for the Care and Use of Laboratory Animals. To evaluate *in vivo* tumor growth, HepG2/vector cells, HepG2/shCOMMD10 cells or HepG2/shCOMMD10/IκBα cells were injected subcutaneously into the left flank of each mouse (5 × 10^6^ cells per mouse and n = 6 per group). Tumors were examined twice weekly; length and width were measured with calipers, and tumor volumes were calculated using the equation (L × W^2^)/2. On day 25, the mice were sacrificed and tumors were sectioned for IHC and hematoxylin–eosin staining.

**Luciferase assay**

COMMD10 overexpression SMMC-7721 cells and COMMD10 deletion HepG2 cells treated with TNFα (10ng/ml) were seeded in triplicate in 24-well plates (3×10^4^/well) overnight. NF-κB luciferase reporter plasmid (1 µg, Genomeditech), 10 ng pGMR-TK *Renilla* plasmid (control luciferase plasmid, Genomeditech), 1 μg COMMD10 and vector plasmid (GeneCopoeia, USA), 1 µl COMMD10 small interfering RNA (siRNA), or negative control siRNA (Suzhou Ribo Life Science) per well were co-transfected into the cells using Lipofectamine 3000 reagent (Invitrogen). At 48 h after transfection, luciferase and *Renilla* activity were measured using a Dual Luciferase Reporter Assay Kit (Promega).

**Co-immunoprecipitation (Co-IP)**

HepG2 cell extracts were incubated 4 h at 4 °C with IgG and protein A/G Agarose to get rid of unspecific binding. COMMD10 antibody (Abcam127691, Cambridge, UK) was then added at 4 °C overnight. The protein A/G-agarose was collected by centrifugation. The beads were suspended in 2×SDS sample buffer and heated to 100 °C for 10 min. Immunoprecipitated proteins were analyzed by western blot.

**GST pull-down assay**

GST-pEGX-6p-1-COMMD10 (1–132a) and (133–202a), Flag, FLAG-p65-WT, FLAG-p65-(1-305), and FLAG-p65-(306-515) plasmids were purchased from HAPK biology (Shenzhen, China). GST-COMMD10 (1–132a), GST-COMMD10(133–202a) and GST were transformed into colibacillus BL21 (DE3) and induced for protein expression by IPTG (0.2mM). Proteins were purified by Pierce^TM^ GST Protein Interaction Pull-Down Kit (Thermo Fisher Scientific, MA, USA）according to the manufacturer’s instructions. After purification, the GST fusion proteins were then incubated with the cell lysates (HEK293T transfected with Flag, FLAG-p65-WT, FLAG-p65-(1-305), and FLAG-p65-(306-515) plasmids, respectively) for 4 h at 4 °C with constant shaking. The beads were washed three times with cold PBS (pH 7.4) and then added with elution buffer (50 mM Tris-Cl,10 mM reduced glutathione, pH 8.0) shaking at 4 °C for 30 min. The eluted proteins were subjected to western blot identification.

**Acridine orange (AO) and ethidium bromide (EB) double staining**

AO and EB (Solarbio) were used for the morphological detection of apoptotic cells. HepG2 cells were transfected in 6-well plates as mentioned above. After 24h, HepG2/COMMD10, HepG2/siCOMMD10, HepG2/siCOMMD10/IκBα cells and corresponding control cells were trypsinized and seeded (2 × 10^4^/well) in 24-well plates overnight, and then treated with cisplatin (10 μg/ml) and incubated for 24 h. The medium was removed and the plate was washed with PBS (pH 7.4). AO/EB cocktail (1:1, 20 µl) was added to 1 ml PBS. After 5-min incubation at room temperature, the plate was observed under a fluorescent microscope (Olympus, ×400 magnification). Green staining indicated live cells; orange and red staining indicated apoptotic and necrotic cells, respectively. The apoptotic index was calculated as the percentage of apoptotic cells from 100 randomly counted cells in each treatment group.

**Caspase 3/7 activation assay**

Cells (3 × 10^4^) were seeded in triplicates in 24-well plate overnight. One microgram COMMD10 and vector plasmid (GeneCopoeia, USA) or 1 μl COMMD10-siRNA and negative control-siRNA (Suzhou Ribo Life Science, Kunshan City, China) per well were transfected into HCC cells using the Lipofectamine^®^ 3000 reagent (Invitrogen). Forty-eight hours after transfection, cells were treated with cisplatin (10 μg/ml) for 24 h. The media was removed, and the plate was washed with fresh media for several times. One microliter of caspase-3/7 solution was added into 1 ml media. After incubation for 30 min at 5% CO_2_ and 37°C, the plate was observed under a fluorescent microscope (Olympus, at ×400). The caspase-3/7 activation fluorescence intensity, which is evaluated by caspase-3/7 positive cells from 5 random sights in each treatment group, was analyzed to calculate apoptotic index by ImageJ software.

**Terminal deoxyribonucleotidyl transferase–mediated dUTP-digoxigenin nick end labeling (TUNEL) assay**

TUNEL assay was carried out using DeadEnd™ Fluorometric TUNEL System (Promega, Madison, USA) according to provided protocol. Briefly, following less washing in Phosphate-buffered saline (PBS) (Sigma-Aldrich, USA), HepG2 cells fixed in 4% paraformaldehyde solution (Wako, Japan) and treated with 0.1% Triton ×100 solution (Sigma, Germany) for 3 min. Then, cells were primarily incubated in TUNEL solution at 37°C for an hour according to the manufacturer's instructions. Negative control was incubated only in fluorescent solution without enzyme to ensure the absence of labeling. For the positive control, a number of cells prior to incubation with TUNEL staining solution were incubated with 50 μg/ml DNase I solution for one hour and then treated with TUNEL solution. Finally, all cells were counterstained with 4; 6-diamidino-2-phenylindole (DAPI) in PBS (Sigma-Aldrich, USA) diluted 1:2 for 10 sec. Then, the number of TUNEL-positive cells was calculated and photographed using a fluorescent microscope (Olympus, ×400 magnification).

**R code for building nomogram**

library(rms)

train0<-read.csv("train.csv",header=T)

train<-train0[!is.na(train0$C10),]

C10<-factor(train$C10)

label(C10)<-"COMMD10"

levels(C10)<-c("Low","High")

size<-factor(train$size)

label(size)<-"size (cm)"

levels(size)<-c("≤5",">5")

ALB<-factor(train$ALB)

label(ALB)<-"ALB (g/L)"

levels(ALB)<-c("≤35",">35")

age<-factor(train$age)

label(age)<-"age (y)"

levels(age)<-c("≤50",">50")

C.embolus<-factor(train$C_embolus)

label(C.embolus)<-"tumor embolus"

levels(C.embolus)<-c("NO","YES")

dd<-datadist(age,size,C10,ALB,C.embolus)

options(datadist='dd')

time<-train$OSmonth

status<-train$Outcome1

f<-cph(Surv(time,status)~age+size+ALB+C.embolus+C10,surv=T)

surv<-Survival(f)

nom<-nomogram(f,lp=F,fun=list(function(x) surv(12,x),

function(x) surv(24,x),

function(x) surv(36,x)),

fun.at=c(.01,0.05,seq(.1,.9,by=.1)),

funlabel=c("1 year survival probability",

"2 year survival probability","3 year survival probability"))

plot(nom) #Output the Nomogram graph

# Output calibration curve in traindata

f1<-cph(Surv(time,status)~age+size+ALB+C.embolus+C10,x=T,y=T,surv=T,time.inc=36)

cal1<-calibrate(f1,cmethod="KM",method="boot",u=36,m=86,B=500)

plot(cal1,lwd=2,lty=1,errbar.col=c(rgb(0,118,192,maxColorValue=255)),xlim=

(0,1),ylim=c(0,1),riskdist=T,subtitles=F,

conf.int=T,xlab="Nomogram-Predicted Probability of 3-Year

Survival",ylab="Actual 3-Year Survival (proportion)",

col=c(rgb(192,98,83,maxColorValue=255)))

lines(cal1[,c("mean.predicted","KM")],type="b",lwd=2,col=c(rgb(192,98,83,m

xColorValue=255)),pch=16)

abline(0,1,lty=3,lwd=2,col=c(rgb(0,118,192,maxColorValue=255)))

validate(f1,B=1000)

**Reference**

1. Hu JL, Wang W, Lan XL, Zeng ZC, Liang YS, Yan YR, Song FY, Wang FF, Zhu XH, Liao WJ, et al: **CAFs secreted exosomes promote metastasis and chemotherapy resistance by enhancing cell stemness and epithelial-mesenchymal transition in colorectal cancer.** *Mol Cancer* 2019, **18:**91.
