# Supplementary material for: COMMD10 inhibits tumor progression and induces apoptosis by blocking NF‐κB signal and values up BCLC staging in predicting overall survival in hepatocellular carcinoma
Source: Clin Transl Med. 2021 May 4;11(5):e403. doi: 10.1002/ctm2.403 (PMC8093973; doi:10.1002/ctm2.403)
Supplement: Supplementary file 2 — SUPPORTING INFORMATION [file CTM2-11-e403-s001.docx]

**Table S1** **The cell proportion of different apoptotic types in AOEB double staining**

| **Groups** | **Normal Rate(%)** | **Early Apoptosis rate (%)** | **Late Apoptosis rate (%)** | **Necrosis rate (%)** | **Apoptosis rate (%)** |
| --- | --- | --- | --- | --- | --- |
| Mock | 58 ± 5 | 27 ± 3 | 14 ± 2 | 1 ± 0 | 41 ± 5 |
| COMMD10+ | 42 ± 5 | 40 ± 2 | 17 ± 4 | 1 ± 0 | 58 ± 4 |
| Vector | 66 ± 3 | 19 ± 3 | 14 ± 3 | 1 ± 1 | 33 ± 3 |
| siCOMMD10 | 86 ± 2 | 9 ± 1 | 5 ± 0 | 1 ± 0 | 13 ± 1 |
| siCOMMD10+IκBα | 70 ± 3 | 20 ± 2 | 10 ± 4 | 1 ± 0 | 29 ± 3 |

HepG2 cells were transfected with indicated plasmid or siRNA for 48h, and then each group treated with cisplatin (10 μg/mL) and incubated for 24 h. The apoptotic index was calculated as the percentage of apoptotic cells from 100 randomly counted cells in each treatment group. Each result represents the mean ±SD.

**Table S2** **The median of 1-, 2-, 3-, and 5-year overall survival in the training and validation cohorts**

| **Factors** | **NF training cohort** | **NF internal validation cohort** | **ZY external validation cohort** |
| --- | --- | --- | --- |
| **1-year OS (95% CI)** | | | |
| Overall | 0.872(0.830,0.916) | 0.886(0.831,0.944) | 0.929(0.883,0.978) |
| COMMD10 low | 0.820(0.751,0.895) | 0.815(0.718,0.926) | 0.885(0.807,0.969) |
| COMMD10 high | 0.919(0.872,0.968) | 0.942(0.888,0.999) | 0.981(0.944,1) |
| **2-year OS (95% CI)** | | | |
| Overall | 0.762(0.707,0.821) | 0.809(0.739,0.885) | 0.874(0.812,0.941) |
| COMMD10 low | 0.692(0.607,0.788) | 0.702(0.585,0.843) | 0.800(0.700,0.915) |
| COMMD10 high | 0.826(0.759,0.899) | 0.891(0.817,0.971) | 0.958(0.902,1) |
| **3-year OS (95% CI)** | | | |
| Overall | 0.740(0.683,0.802) | 0.712(0.630,0.805) | 0.826(0.752,0.906) |
| COMMD10 low | 0.657(0.570,0.758) | 0.540(0.409,0.714) | 0.731(0.618,0.866) |
| COMMD10 high | 0.815(0.746,0.890) | 0.836(0.748,0.935) | 0.931(0.859,1) |
| **5-year OS (95% CI)** | | | |
| Overall | 0.660(0.587,0.743) | 0.641(0.544,0.755) | 0.810(0.733,0.895) |
| COMMD10 low | 0.496(0.375,0.656) | 0.363(0.208,0.634) | 0.703(0.585,0.846) |
| COMMD10 high | 0.795(0.719,0.880) | 0.815(0.721,0.922) | - |

**Table S3** **The** **univariate analysis in the NF training cohort**

| Characteristics | HR (95%CI) | *P* value |
| --- | --- | --- |
| COMMD10 | 0.45 (0.27-0.73) | 0.001 |
| Gender | 0.80 (0.35-1.85) | 0.601 |
| Age(years) (>50 versus ≤50) | 1.86 (1.13-3.06) | 0.014 |
| Smoking | 1.07 (0.68-1.68) | 0.78 |
| Drinking | 1.18 (0.74-1.88) | 0.498 |
| Symptom | 2.02 (1.24-3.29) | 0.005 |
| Tumor differentiation | 1.41 (1.02-2.15) | 0.045 |
| Tumor number | 1.88 (1.14-3.1) | 0.013 |
| Tumor size(cm) (>5 versus ≤5) | 2.20 (1.36-3.57) | 0.001 |
| Satellite focus | 2.06 (1.08-3.93) | 0.029 |
| MVI | 2.64 (1.41-4.95) | 0.002 |
| Vascular invasion | 2.82 (1.67-4.78) | <0.001 |
| Tumor embolus | 4.02 (2.21-7.29) | <0.001 |
| Ascites | 1.57 (0.89-2.77) | 0.117 |
| CP grade | 2.06 (1.03-4.15) | 0.042 |
| Lymph nodes metastasis | 2.61 (1.05-6.5) | 0.039 |
| BCLC stage | 2.77 (1.66-4.61) | <0.001 |
| HBsAg | 0.9 (0.48-1.72) | 0.757 |
| HBs Ab | 1.17 (0.6-2.28) | 0.648 |
| HBe Ag | 0.74 (0.34-1.62) | 0.454 |
| HBe Ab | 0.99 (0.6-1.63) | 0.958 |
| HBc Ab | 0.8 (0.4-1.6) | 0.521 |
| Cirrhosis | 1.29 (0.75-2.24) | 0.358 |
| Fatty liver | 0.95 (0.38-2.37) | 0.921 |
| PHT | 1.61 (0.9-2.88) | 0.107 |
| Hepatitis | 0.79 (0.43-1.53) | 0.810 |
| Hypertension | 0.49 (0.22-1.06) | 0.071 |
| Diabetes | 0.64 (0.26-1.59) | 0.336 |
| Family history of cancer | 1.66 (1.07-2.56) | 0.023 |
| AFP (μg/L)(≥400 versus <400) | 1.53 (0.95-2.46) | 0.083 |
| CA199 (U/mL)(≥37 versus <37) | 1.83 (0.73-4.58) | 0.199 |
| ALT(U/L)(≥40 versus <40) | 1.1 (0.69-1.76) | 0.687 |
| AST(U/L)(≥40 versus <40) | 1.96 (1.24-3.07) | 0.004 |
| ALB (g/L)(≥35 versus <35) | 0.42 (0.25-0.70) | 0.001 |
| TBIL(μmol/L)(>17.1 versus ≤17.1) | 1.04 (0.62-1.74) | 0.878 |
| PT(s)(>13 versus ≤13) | 1.66 (1.05-2.63) | 0.031 |
| Adjuvant treatment | 0.91 (0.57-1.46) | 0.689 |
| TACE | 1.2 (0.75-1.91) | 0.448 |
| RFA | 0.7 (0.26-1.93) | 0.494 |
| Target therapy | 2.47 (1.06-5.77) | 0.037 |
| Biotherapy | 1.2 (0.29-4.91) | 0.799 |
| Chemotherapy | 0.61 (0.19-1.98) | 0.415 |
| Reoperation | 0.19 (0.05-0.76) | 0.019 |

MVI = microvascular invasion; CP=Child-Pugh HBV = hepatitis B virus; HCV = hepatitis C virus; PHT = portal hypertensive gastropathy; AFP = α-fetoprotein; ALT =Alanine aminotransferase; AST = Aspartate aminotransferase; TBIL = total bilirubin; ALB = Albumin; PT = Prothrombin time; BCLC stage = Barcelona Clinic Liver Cancer stage; TACE = transcatheter arterial chemoembolization; RFA = Radiofrequency ablation; CI = confidence interval.

**Table S4** **The** **multivariate analysis in the NF training cohort**

| **Variable** | **HR(95%CI)** | ***P* value** |
| --- | --- | --- |
| COMMD10 | 0.524(0.315,0.87) | 0.013 |
| Age | 1.861(1.118,3.099) | 0.017 |
| Tumor size | 2.021(1.187,3.441) | 0.010 |
| Tumor embolus | 3.341(1.777,6.279) | <0.001 |
| ALB | 0.414(0.24,0.711) | 0.001 |

**Table S5: The concordance index of the nomogram and Barcelona Clinic Liver Cancer stage for the prediction of overall survival in the training and validation cohorts**

| **Factor** | **NF training cohort** | | **NF internal validation cohort** | | **ZY external validation cohort** | |
| --- | --- | --- | --- | --- | --- | --- |
|  | **C-index (95% CI)** | ***p*^﹟^** | **C-index (95% CI)** | ***p*^﹟^** | **C-index (95% CI)** | ***p*^﹟^** |
| COMMD10 | 0.592（0.530-0.655） | - | 0.659（0.581-0.736） | - | 0.664（0.570-0.758） | - |
| Nomogram | 0.716（0.648-0.783） | - | 0.796（0.726-0.866） | - | 0.720（0.600-0.840） | - |
| BCLC stage | 0.635（0.577-0.693） | - | 0.606（0.530-0.681） | - | 0.544（0.388-0.699） | - |
| COMMD10 vs. Nomogram stage | - | <0.001 | - | <0.001 | - | 0.216 |
| COMMD10 vs. BCLC stage | - | 0.133 | - | 0.172 | - | 0.038 |
| Nomogram vs. BCLC stage | - | 0.013 | - | <0.001 | - | 0.007 |

﹡Nomogram included six factors (COMMD10,age, size, tumor embolus, tumor differentiation, and Albumin).

﹟*p* values were calculated based on normal approximation using the rcorrp.cens function of the Hmisc package.

C-index = concordance index; CI = confidence interval; BCLC stage = Barcelona Clinic Liver Cancer stage.
