# Supplementary material for: COMMD10 inhibits tumor progression and induces apoptosis by blocking NF‐κB signal and values up BCLC staging in predicting overall survival in hepatocellular carcinoma
Source: Clin Transl Med. 2021 May 4;11(5):e403. doi: 10.1002/ctm2.403 (PMC8093973; doi:10.1002/ctm2.403)
Supplement: Supplementary file 3 — SUPPORTING INFORMATION [file CTM2-11-e403-s003.docx]

**COMMD10 inhibits tumor progression and induces apoptosis by blocking NF-κB signal and values up BCLC staging in predicting overall survival in hepatocellular carcinoma**

Mi Yang^1, *^, Xixi Wu^1, *^, Lu Li^1, *^, Shaoqun Li^1^, Nan Li^1^, Mengyuan^1^ Mao, Suming Pan^2^, Richang Du^3^, Xiaoqing Wang^1^, Min Chen^1^, Nanjie Xiao^1^, Xiaohui Zhu^4, 5, 6^, Guoyang He^4, 5, 6^, Longshan Zhang^1^, Weiqiang Huang^1^, Hua Pan^1^, Lan Deng^7^, Longhua Chen^1, #^, Li Liang^4, 5, 6, #^, Jian Guan^1,^ ^#^

**Supplementary Figures**

**
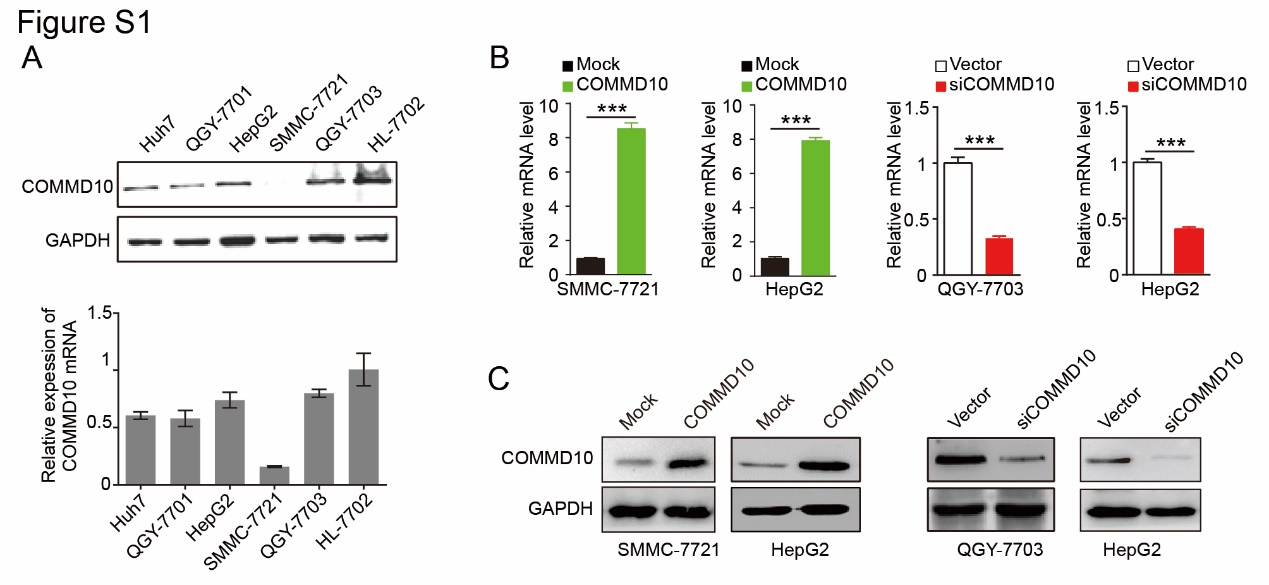
**

**Figure S1. COMMD10 expression and transfection efficiency in HCC cells transfected with COMMD10 overexpression plasmid or siCOMMD10 plasmid.** (A) COMMD10 protein (top) and mRNA levels (bottom) in HCC cell lines and normal HL-7702 cell line; (B-C) COMMD10 transfection efficiency was validated by qPCR (B) and western blot (C); GAPDH was used as the loading control. Each bar represents the mean ± SD; ****P* < 0.001.


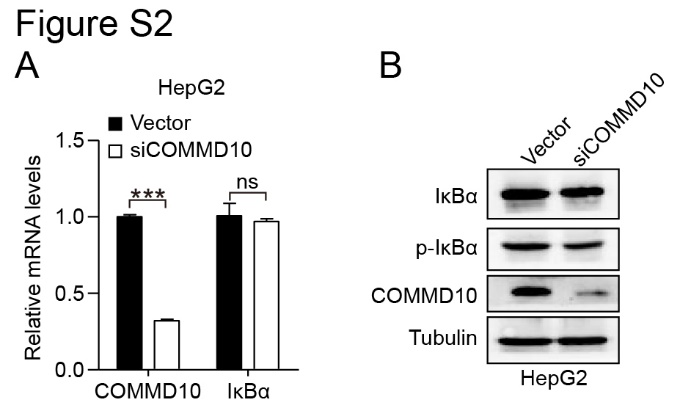


**Figure S2. Effect of COMMD10 deficiency on the level of IκBα mRNA (A) and protein (B) in HepG2 cells.**


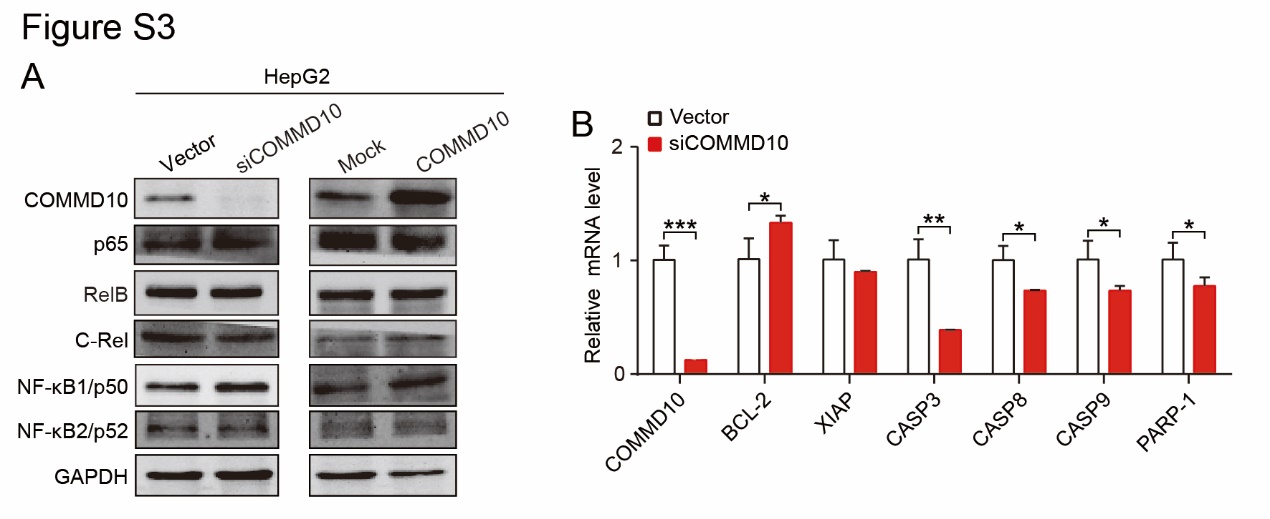


**Figure S3. Expression of NF-κB family members and downstream genes in ectopic COMMD10 HepG2 cells.** (A) Effect of COMMD10 on the expression of NF-κB subunits in HepG2 cells by Western blot. GAPDH was used as internal control; (B) Real-time PCR examination of NF-κB–regulated apoptosis-related genes in COMMD10-depleted HepG2 cells. Each bar represents the mean ± SD; **P* < 0.05, ***P* < 0.01, ****P* < 0.001.

**
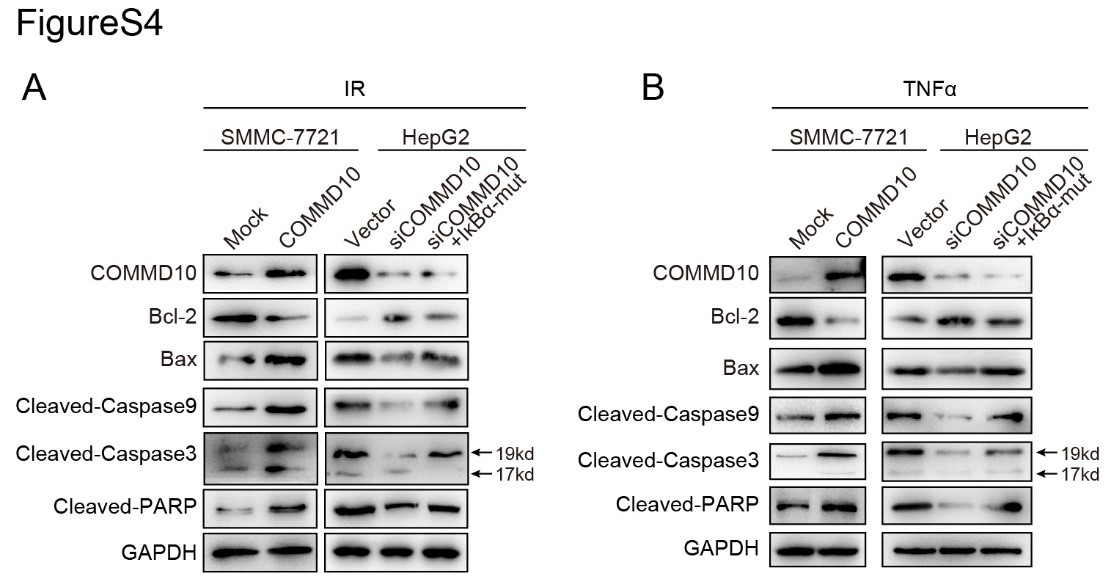
**

**Figure S4. Expression of Bcl2, Bax, cleaved-caspase3, cleaved-caspase9, cleaved-PARP in the indicated cells exposed to radiation (6Gy)(A) or TNFα (10ng/mL)(B). GAPDH was used as a loading control.**

**
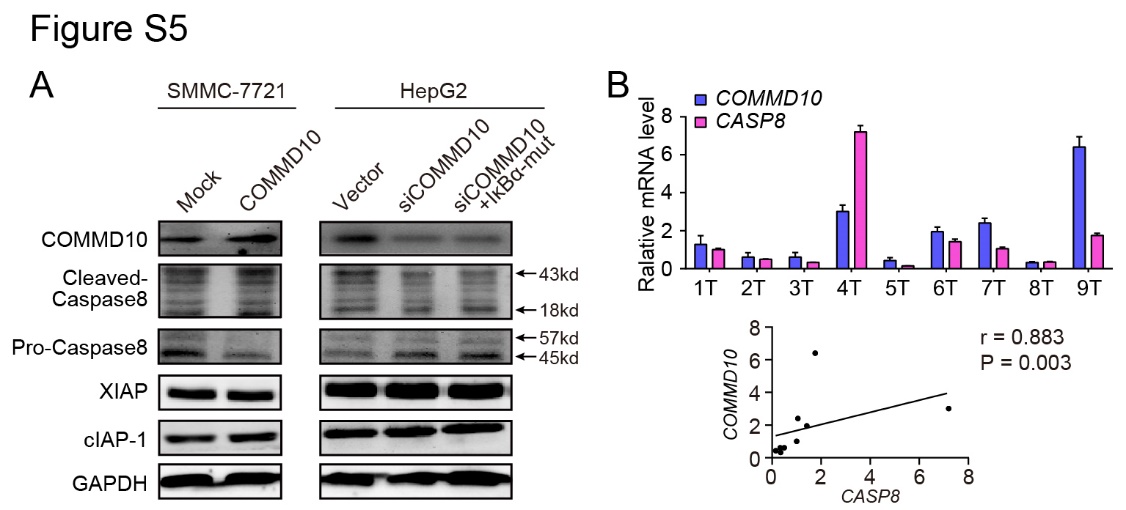
**

**Figure S5. Expression of apoptosis-associated genes in ectopic COMMD10 cells and correlation of COMMD10 with CASP8 in HCC tissues.** (A) Western blot examination of the expression of apoptosis-related genes in indicated SMMC-7721 and HepG2 cells; (B) Relative expression and correlation of COMMD10 and CASP8 mRNA in HCC tissues.


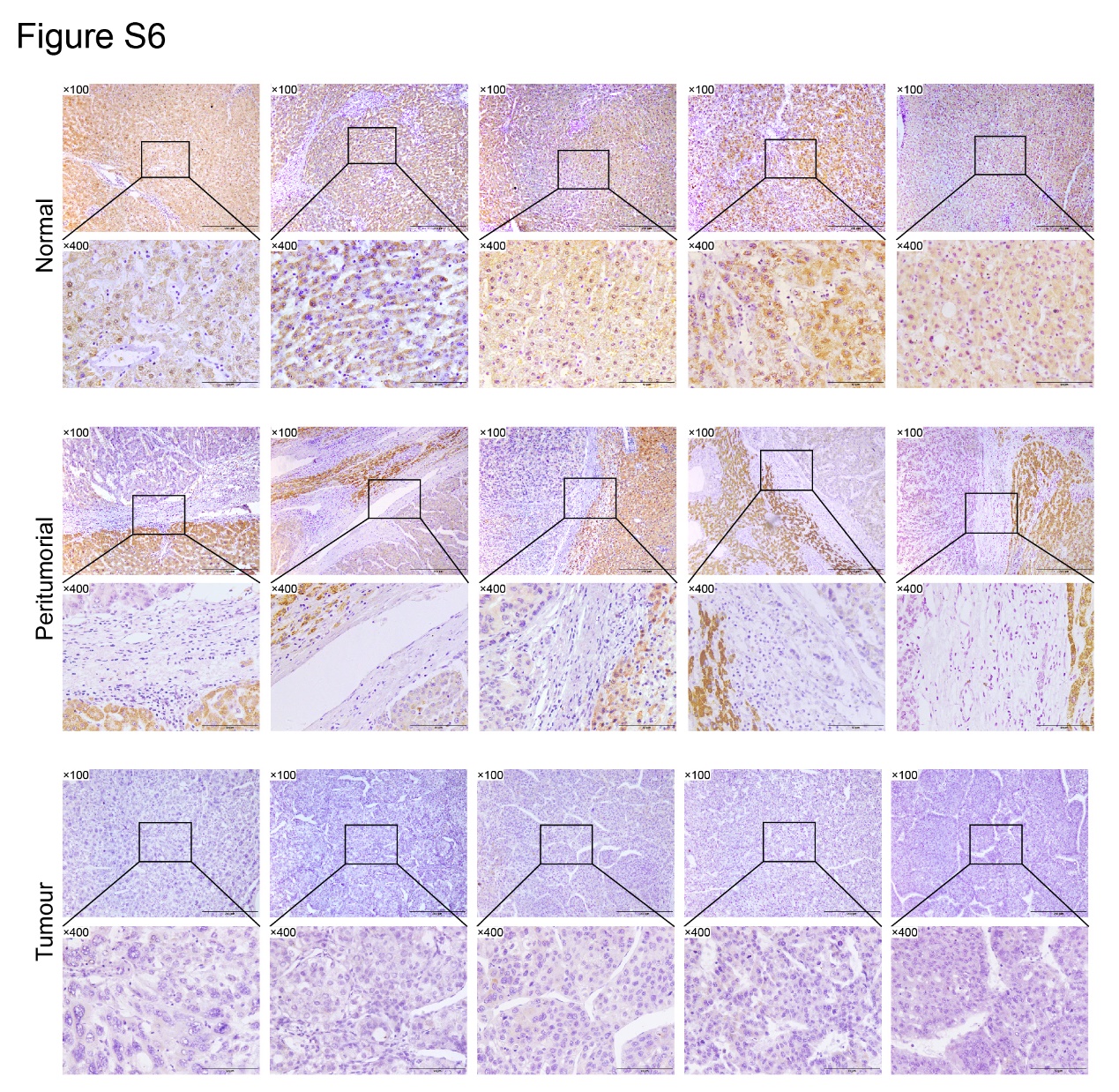


**Figure S6. Strong expression of COMMD10 protein in paraffin-embedded normal, peritumorial liver tissues and weak expression of COMMD10 in paraffin-embedded HCC tissues by IHC staining analysis.**


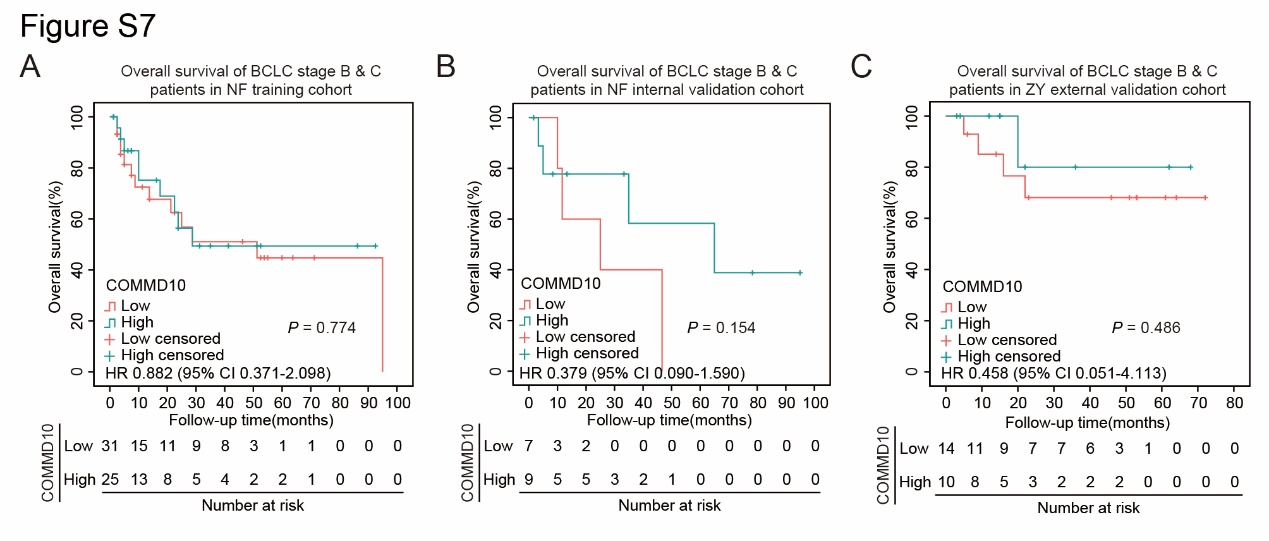


**Figure S7. Prediction of advanced HCC patients’ OS based on COMMD10 expression.** (A-C) Prediction of advanced HCC (BCLC stage B and C) patients’ OS based on COMMD10 expression in NF training cohort (A), NF internal validation cohort (B), and ZY external validation cohort (C) using Kaplan-Meier survival analysis.


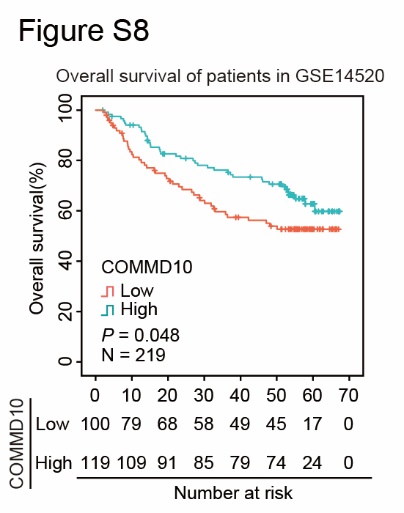


**Figure S8. Prediction of HCC patients’ OS based on COMMD10 expression in GSE14520 data**
